# A standardized framework resolves ambiguity in motor neuron loss across neurodegenerative diseases

**DOI:** 10.1186/s40478-026-02415-7

**Published:** 2026-09-01

**Authors:** Leonie Sowoidnich, Aaron L. Norman, Florian Gerstner, Josiane K. Siemund, Jannik M. Buettner, John G. Pagiazitis, Vanessa Dreilich, Konstantin Pilz, Dajun Tian, Charlotte J. Sumner, Angela Paradis, George Z. Mentis, Christian M. Simon

**Affiliations:** 1 Carl-Ludwig-Institute for Physiology, Leipzig University, 04103 Leipzig, Germany; 2 Center for Motor Neuron Biology and Disease, Columbia University, New York, NY 10032 USA; 3 Depts. of Pathology and Cell Biology and Neurology, Columbia University, New York, NY 10032 USA; 4 Biogen, Cambridge, MA 02142 USA; 5 Department of Neurology, Johns Hopkins University School of Medicine, Baltimore, MD 21205 USA; 6 Department of Neuroscience, Johns Hopkins University School of Medicine, Baltimore, MD 21205 USA; 7 Department of Genetic Medicine, Johns Hopkins University School of Medicine, Baltimore, MD 21218 USA

**Keywords:** Motor neuron quantification, Neurodegeneration, Motor neuron diseases, Spinal muscular atrophy, Amyotrophic lateral sclerosis, Spinal cord, SMN∆7, SOD1-G93A, 4-copy SMN2 Type III-like SMA

## Abstract

Motor neuron (MN) loss is a hallmark of neurodegenerative disorders, yet its assessment remains variable, confounding mechanistic and therapeutic interpretation. To address this, we conducted a systematic review and meta-analysis of spinal muscular atrophy (SMA) mouse studies, revealing 60% variability in reported MN loss, for which spinal cord sampling emerged as a major contributor. Using a whole-segment approach with tissue clearing, MN tracing, and multimodal imaging, we confirmed segment-dependent differences in MN counts. Common MN markers (SMI-32, Nissl) lacked specificity, whereas choline acetyltransferase (ChAT) provided robust labeling in murine and human spinal cords. Deep learning–based whole-mount segmentation enabled unbiased MN quantification and validated manual counts. Integrating analysis with computational modeling established segment sampling as a key driver of variability and revealed degeneration patterns: widespread MN loss in amyotrophic lateral sclerosis (ALS), selective MN loss in severe SMA, and preservation in mild SMA models. These findings establish a framework for reproducible MN quantification.

**Supplementary Information:**

The online version contains supplementary material available at https://doi.org/10.1186/s40478-026-02415-7.

## Highlights

- Spinal cord segment-specific analysis reduces variability and allows accurate MN quantification.
- ChAT is the most reliable MN marker in murine and human spinal cords.
- Deep learning–based segmentation enables unbiased MN quantification in intact spinal cords.
- MN degeneration is widespread in ALS but restricted to pools innervating proximal muscles in severe SMA.

**Supplementary Information:**

The online version contains supplementary material available at https://doi.org/10.1186/s40478-026-02415-7.

## Introduction

Spinal motor neurons (MNs) constitute the final common pathway of the motor system, driving essential functions including posture, locomotion, speech, and breathing. Their degeneration results in severe motor deficits, paralysis, and ultimately death [1–3]. MN loss occurs physiologically during embryonic development as part of programmed cell death [4, 5] but can also arise after trauma, ischemia, inflammation, as well as in neurodegenerative disorders such as poliomyelitis, hereditary motor neuropathies, and progressive muscular atrophy [6–10]. MN degeneration is also a defining feature of MN diseases, including spinal muscular atrophy (SMA), amyotrophic lateral sclerosis (ALS), spinal and bulbar muscular atrophy (SBMA), and spinal muscular atrophy with respiratory distress type 1 (SMARD1) [11–14]. Accurate and reproducible MN quantification is therefore essential for understanding the mechanisms governing MN survival and degeneration across development, injury, and disease.

Owing to the limited availability, variable quality and genetic heterogeneity of human postmortem tissue, mouse models are indispensable for longitudinal and mechanistic studies of MN degeneration. Several mouse models reliably recapitulate key features of disease and enable quantitative assessment of MN loss as a robust readout of disease severity, progression, and therapeutic response. However, the reported extent of MN loss varies widely even within the same disease mouse models [15–20], highlighting methodological variability and raising concerns about reproducibility, cross-study comparisons, and the interpretation of mechanistic and therapeutic outcomes.

A major source of variability may stem from inconsistent sampling of spinal cord regions containing disease-specific vulnerable versus resistant MN pools. The longitudinal organization of MNs into columns that innervate distinct muscle groups [21–24] underlies characteristic segmental vulnerabilities, with distal muscles preferentially affected in ALS and proximal muscles in SMA [11, 25–28]. Consequently, analyses targeting different spinal segments or MN pools may yield markedly different estimates of MN loss.

Beyond anatomical sampling, the molecular markers used to identify MNs represent another important source of variability in MN quantification**.** Classical Nissl and hematoxylin–eosin (H&E) staining have long been used to identify large ventral horn neurons presumed to be MNs but lack specificity, as they also label large non-MN neuronal populations within the ventral horn [29–31]. SMI-32, an antibody recognizing non-phosphorylated neurofilament H, has also been utilized as an MN marker in rodents and humans, although accumulating evidence indicates expression in other ventral horn neuronal populations [32–34]. More selective markers, including choline acetyltransferase (ChAT) and the homeobox protein HB9 are widely used but also label subsets of spinal interneurons outside MN pools [35–37], potentially confounding MN identification and the interpretation of experimental outcomes [38, 39]. Together, these limitations underscore the need for a systematic and quantitative comparison of commonly used MN markers to minimize errors in MN quantification.

Quantification strategies represent an additional source of variability in estimates of MN loss. While stereology-like methods aim to provide unbiased estimates of MN number, they require strict sampling parameters and are sensitive to tissue processing artifacts. Conversely, manual counting approaches are time-consuming, labor-intensive, and prone to inter-observer variability. Both approaches can be affected by developmental or disease-related changes in spinal cord size and morphology [39]. Collectively, these challenges highlight the need to identify sources of variability and establish standardized approaches for MN quantification.

To address these issues, we performed a systematic literature review (SLR) and meta-analysis across SMA mouse models, identifying spinal cord segment selection as a major source of inter-study variability. We developed a standardized framework for reliable, reproducible, and unbiased MN quantification. To this end, we combined retrograde labeling with ChAT immunostaining, validated candidate MN-specific markers, assessed developmental and tissue-processing effects, and established both manual and machine-learning–based quantification approaches. Applying this framework revealed segment- and column-specific MN numbers in wild-type mice, as well as distinct selective MN vulnerabilities in ALS and SMA mouse models. Together, this study establishes a detailed, robust and scalable framework for standardized MN quantification across experimental paradigms and disease models.

## Results

### Meta-analysis identifies spinal segment sampling as a major contributor to variability in reported motor neuron loss in SMA

**Fig. 1:**
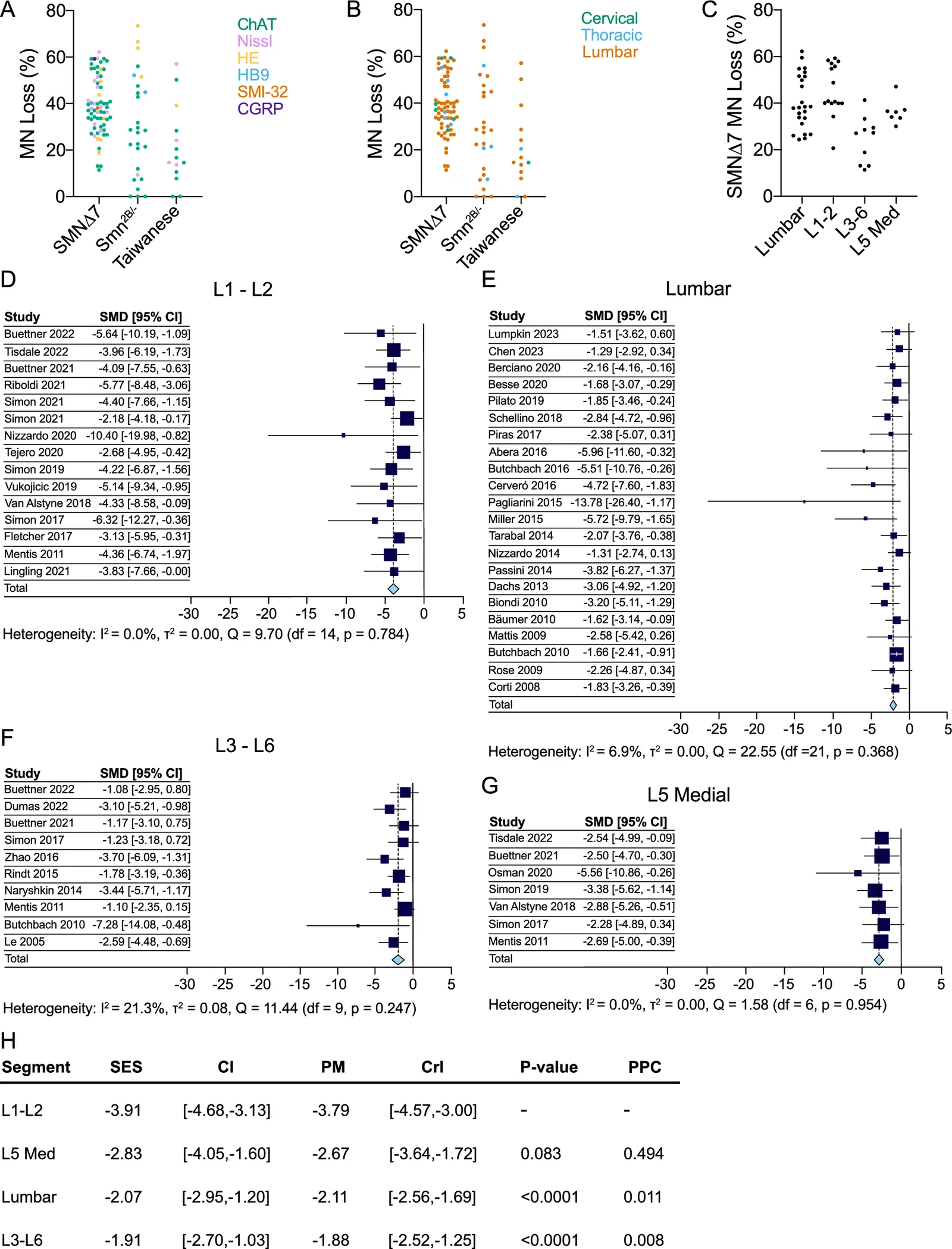
SLR and subgroup meta-analysis reveal spinal segment-dependent variability in MN loss in SMA mouse models. MN loss (%) across studies included in the SLR for three SMA models, SMNΔ7, Smn^2B/–^ and Taiwanese, grouped by MN marker **A** or spinal cord region analyzed **B**. **C** MN loss (%) in the lumbar spinal cord of SMNΔ7 mice, subdivided into lumbar (segment not specified in the included studies), L1–L2, L3–L6 and L5 Medial. Summary of subgroup analyses for studies using the SMNΔ7 mouse model: L1–L2 **D**, lumbar **E**, L3–L6 **F** and L5 Medial **G**. Blue boxes and black bars represent effect sizes and 95% confidence intervals, respectively. Larger boxes indicate greater study weight. Dotted lines indicate the summary effect size for each subgroup. SMD, standardized mean difference, a statistical measure equivalent to effect size; all subsequent references to effect size refer to this metric. I^2^, estimated heterogeneity; τ^2^, between-study variance in true effect size; Q (Cochran’s Q), heterogeneity statistic. **H** Comparison of subgroup-analysis estimates with a Bayesian model. SES, summary effect size; CI, 95% confidence interval; PM, posterior mean; CrI, credible interval; PC, pairwise comparison; PPC, posterior probability of contrast

Individual studies diverge markedly in the extent of reported MN loss, even when using identical SMA mouse models [15–20]. To obtain an unbiased overview of this variability, we performed an SLR of SMA studies indexed in PubMed (Table S1). Using predefined population, intervention, comparator, outcomes, and study type (PICOS) criteria (Table S2), we identified 75 publications with data from the three most commonly used SMA mouse models: SMN∆7, Smn^2B/–^, and Taiwanese (Table S3 and Table S6). The SMN∆7 model was most frequently used (65% of studies), whereas Smn^2B/–^ and Taiwanese models accounted for ~ 23% and 12% respectively (Fig. S1A). Across studies, the lumbar spinal cord was the most frequently examined region (82.5%), followed by the thoracic (11%) and cervical (6.5%) segments (Fig. S1B). ChAT (57%) immunostaining was the predominant method for MN quantification, with Nissl staining (20%), H&E (18%), HB9 (3%), SMI-32 and calcitonin gene-related peptide (CGRP) (1% each) being used less frequently (Fig. S1C). Remarkably, in these studies, reported MN loss varied widely (range: 0–60%) across all three models, and this variability persisted irrespective of the MN marker used or the spinal region analyzed (Fig. 1A, B). To determine whether this disparity in MN loss persists within a single spinal region, we further examined the lumbar spinal cord of SMNΔ7 mice, the most frequently studied experimental context. The depth of data gathered within this model alone facilitated the subdivision of lumbar MNs into pools innervating vulnerable proximal muscles (L1–L2 and the medial motor column at L5) and pools innervating more resistant distal muscles (L3–L6) [40]. Studies that did not specify segmental levels were classified as general lumbar (Fig. 1C). This analysis revealed striking variability in reported MN loss (Fig. 1C), exposing potentially major inconsistencies in the SMA literature.

We next wanted to address the nature of the marked variability in MN loss observed in the SLR by performing a meta-analysis restricted to the lumbar spinal cord of the SMN∆7 mouse model, integrating 43 publications comprising 54 lumbar region data groupings. The effect sizes derived from control and SMA MN counts showed low overall heterogeneity (I^2^ = 16.2%, Q(df=53) = 66.0499, *p* = 0.1075), though the bootstrapped upper 95% confidence interval bound approached 51.5%, suggesting moderate heterogeneity. We next considered which factors could be acting as confounders hindering study comparability. Immunohistochemical staining and quantification methods presented a significant source of variability between studies, but aspects regarding staining quality and marker combinations prevented further consideration in this analysis. In the same light, methodologies in obtaining spinal sections varied dramatically in thickness, space between sections, criteria for identifying MNs and the number of sections used so that no unified grouping could be formed. Genetic drift and conditions of murine upkeep also presented a limitation for which sufficient data could not be gathered. Furthermore, life span of SMNΔ7 mice is heterogeneous and severe MN death and motor impairment occur after one week [41]. For this reason, we defined end-stage starting at P7. Another factor that varied among the reporting was the spinal segment. This could be controlled for in the subsequent subgroup analysis by spinal segment (Lumbar, L1–L2, L3–L6, L5 medial motor columns (MMC)), which revealed a significantly improved model fit (Q(3) = 19.55, *p* = 0.0002; Fig. S1F) and markedly reduced intra-study heterogeneity (Fig. 1D–G). The test of moderators of this model was significant (I^2^ = 0.0%, Q(M)(df=3) = 20.7736, *p* = 0.0001), indicating that spinal region accounted for a substantial proportion of the between-study heterogeneity. Segment-specific analyses confirmed minimal overlap of the effect size confidence intervals across groups (Fig. 1H), indicating a unique relation between MN loss and spinal segment. Furthermore, Bayesian multilevel modeling with segment identity as a control reinforced these findings by revealing comparatively narrowed credible intervals (Fig. S1E). Additional analyses with pairwise comparisons and Bayesian posterior probabilities showed that “L1–L2” contained the strongest relation to MN loss, clearly distinct from either “Lumbar” or “L3–L6 segments”, whereas L5 MMC exhibited a weaker but consistent trend (Fig. 1H, S1D–E).

Together, meta-analytic and Bayesian analyses identify L1–L2 MN pools as the most vulnerable and reveal segmental sampling as a key driver of variability in reported MN loss across SMA studies.

### Divergent motor neuron distribution across the lumbar spinal cord revealed by segment-specific analysis

**Fig. 2:**
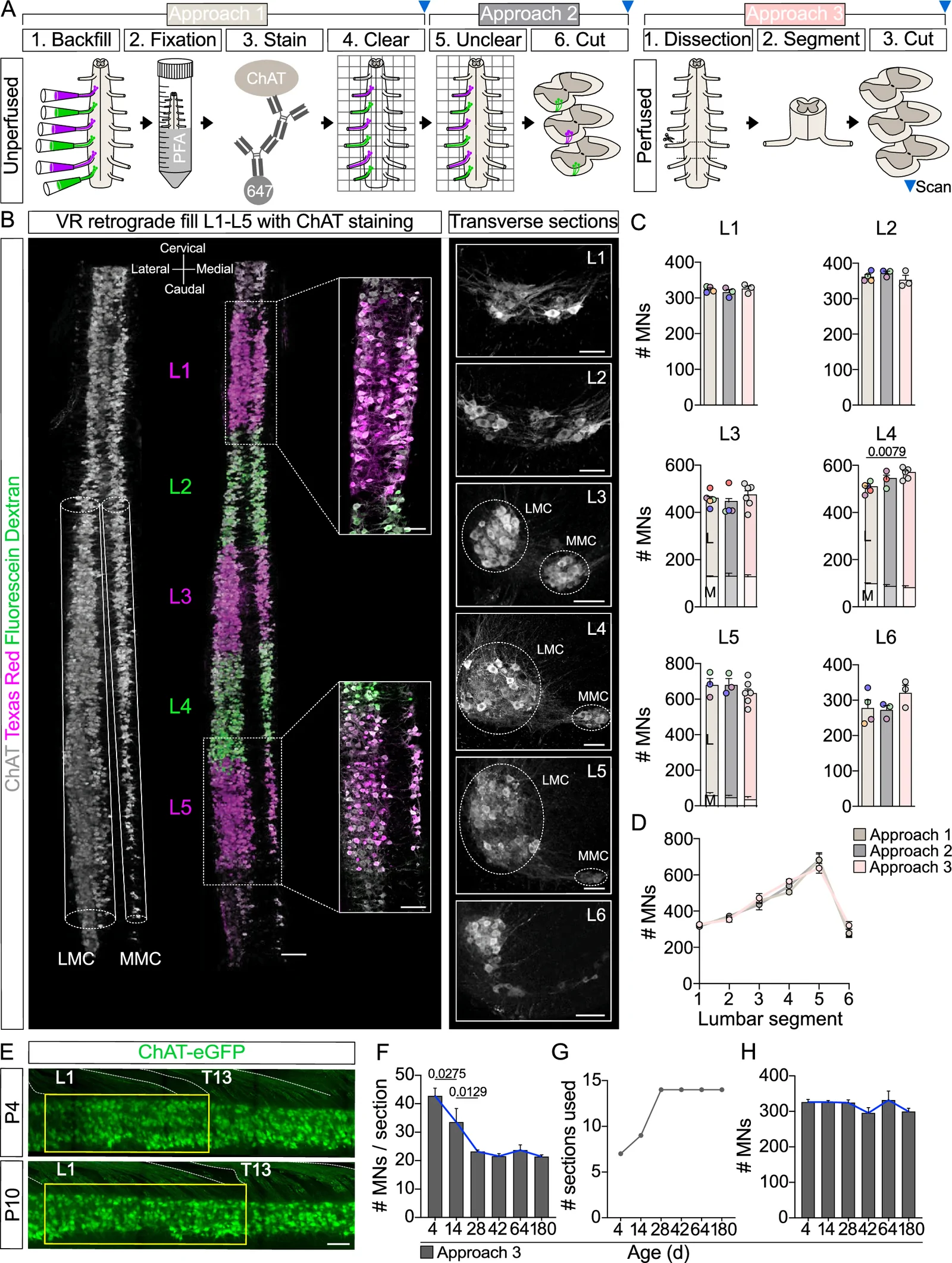
Sectioning-based workflow enables reproducible, segment-specific quantification of lumbar MNs. **A** Schematic of the clearing workflow (Approaches 1 and 2) and dissection workflow (Approach 3). Blue arrows indicate the imaging time point within each approach. **B** Confocal image of a ChAT-stained (gray), cleared intact lumbar spinal cord from a P4 C57BL/6 mouse with retrogradely labeled segments in alternating order of Texas Red (magenta) and Fluorescein Dextran (green). Scale bar = 200 µm. Highlighted insets show higher magnification views of L1 and L5 segments. Transverse sections of ChAT-stained spinal cord show individual lumbar segments, with the MMC and LMC indicated. Scale bars = 50 µm. **C** Quantification of MNs in L1–L6 of the intact lumbar spinal cord (Approach 1), vibratome-sectioned segments of the same spinal cord identified by retrograde labeling (Approach 2), and sections of individual spinal cord segments identified by ventral roots (Approach 3). Colored points represent individual animals, with matching colors indicating the same animal across Approaches 1 and 2. For each segment, n values are listed in the order of Approach 1, Approach 2, and Approach 3: L1: n = 4, 3, 3; L2: n = 4, 3, 3; L3: n = 5, 4, 6; L4: n = 4, 3, 5; L5: n = 3, 3, 6; L6: n = 4, 3, 3. L (LMC) and M (MMC) denote MN counts from the respective motor columns. **D** Quantification of MNs from the same groups as in **C**, plotted across lumbar spinal segments. For segments L3–L5, LMC and MMC counts were combined. **E** T13 to L1 MN pool from a cleared whole-mount spinal cord of P4 and P10 ChAT-eGFP mice. T13 and L1 ventral roots are outlined in white. The yellow box indicates the L1 spinal cord segment as defined by ventral root position. Scale bar = 100 µm. **F** Number of MNs per vibratome Sect. (75 µm), **G** number of vibratome sections spanning the entire L1 segment, and **H** total number of MNs in the L1 segment from C57BL/6 mice at P4, P14, P28, P42, and P180, as identified by ventral root dissection (Approach 3). P4 and P180 data points are from Fig. 2C and S6K. (n = 3 unless otherwise indicated; P180, n = 4). Statistical analysis was performed using one-way ANOVA (C: L1, L2, L4, L5; F, H) and Kruskal–Wallis test (C: L3) comparing the total number of MNs

To translate our meta-analysis findings into experimental validation, we first sought to establish a reliable, segment-specific method for quantifying MNs in spinal cord tissue. To map segmental variation in MN numbers, we dissected spinal cords from four-day-old (P4) C57BL/6 mice and retrogradely filled L1–L6 ventral roots ex vivo with the fluorescent dyes Fluorescein Dextran and Texas Red in an alternating manner (Fig. 2A and Fig. S2A, C, D). Spinal cords were subsequently immersion-fixed with 4% paraformaldehyde (PFA), ChAT-immunostained, cleared (Fig. S2B), and imaged in whole-mount (the intact spinal cord without any sectioning) by confocal microscopy (Approach 1, Fig. 2A, B). To directly compare MN quantification in intact spinal cords and sections, we washed these cleared lumbar spinal cords in PBS after the first imaging session to reverse clearing, sectioned them into 75 µm-thick transverse slices using a vibratome, and subsequently re-imaged them (Approach 2, Fig. 2A, B). Retrograde ex vivo labeling in whole-mount and sectioned preparations revealed minimal rostro–caudal overlap between adjacent MN pools, restricted to single sections, enabling precise assignment of MNs to specific segments (Fig. 2B and Fig. S2D). While 60% of ChAT⁺ MNs were retrogradely filled, nearly all (98%) labeled MNs were ChAT⁺, confirming the specificity of ventral root retrograde labeling and validating ChAT immunoreactivity as a reliable marker of MN identity (Fig. S2E-G). Along the span of L2, MNs segregated into lateral motor columns (LMC) and MMC, with LMC numbers increasing and MMC numbers decreasing along the rostro-caudal axis with the exception of L6, in which MNs formed small, discrete pools rather than large continuous columns (Fig. 2B, C). Manual segmental analysis showed MN numbers increasing from ~ 300 in the rostral lumbar segment L1 to ~ 700 the in caudal lumbar segment L5 before declining to ~ 300 in L6 in both approaches (Fig. 2B–D). These consistent MN counts in whole-mount and sectioned cords validate the retrograde spinal root-labeling with ChAT as a reliable quantification method and demonstrate that lumbar sections cannot be pooled together due to segment-specific differences.

To validate a less labor-intensive approach for segment-specific quantification without retrograde labeling [20, 26, 42, 43], we perfused P4 C57BL/6 mice and dissected lumbar segments using ventral root landmarks as described previously [44] (Fig. S2A). Each segment was sectioned in its entirety at 75 µm thickness with a vibratome, stained for ChAT, and analyzed for MNs by confocal microscopy (Approach 3, Fig. 2A). MN numbers of each segment were found to be indistinguishable between Approaches 1 and 2, establishing this sectional method as a practical and reproducible tool for routine, segment-specific MN quantification (Fig. 2C, D).

To evaluate the reliability of this method throughout the spinal cord development and to determine the impact of maturation upon MN column dimensions, we cleared perfused whole spinal cords from P4 and P10 mice, which expressed eGFP under the ChAT promoter (ChAT-eGFP). Confocal scans of the whole-mount T13–L2 region suggested that total MN numbers remained stable between P4 and P10, but, as the L1 segment lengthened during postnatal development, MN density decreased (Fig. 2E). To quantify these differences, we compared MN numbers per section, MNs per segment, and sections per segment in ChAT-stained and retrogradely labeled L1 segments of FVB control mice of the SMNΔ7 mouse line (Fig. S2I–K). While the number of MNs per section decreased from P4 to P13 (Fig. S2I), the length of the developing spinal segment increased, as reflected by a higher number of sections per segment (Fig. S2J), resulting in stable MN numbers across ages (Fig. S2K). A similar–though slightly delayed–trend was observed in C57BL/6 mice when L1 segments were delineated using ventral root landmarks (Approach 3). Here, MN numbers per section decreased from P4 through P14–P28, accompanied by increasing segmental length, but reached a plateau at later time points (Fig. 2F–H), indicating completion of the maturation process after the fourth postnatal week. Importantly, the total number of MNs per L1 segment was consistent across ages (Fig. 2H and Fig. S2K), demonstrating that the quantification of all MNs within an entire spinal segment provides a reliable metric across developmental stages.

Overall, whole-mount spinal cord analysis and retrograde tracing reveal a rostro-caudal gradient in lumbar MN numbers and validate segment-specific ChAT-based quantification as a practical, reliable, and reproducible approach for routine MN analysis.

### Spinal interneurons undermine the specificity of SMI-32 and Nissl as motor neuron markers

**Fig. 3:**
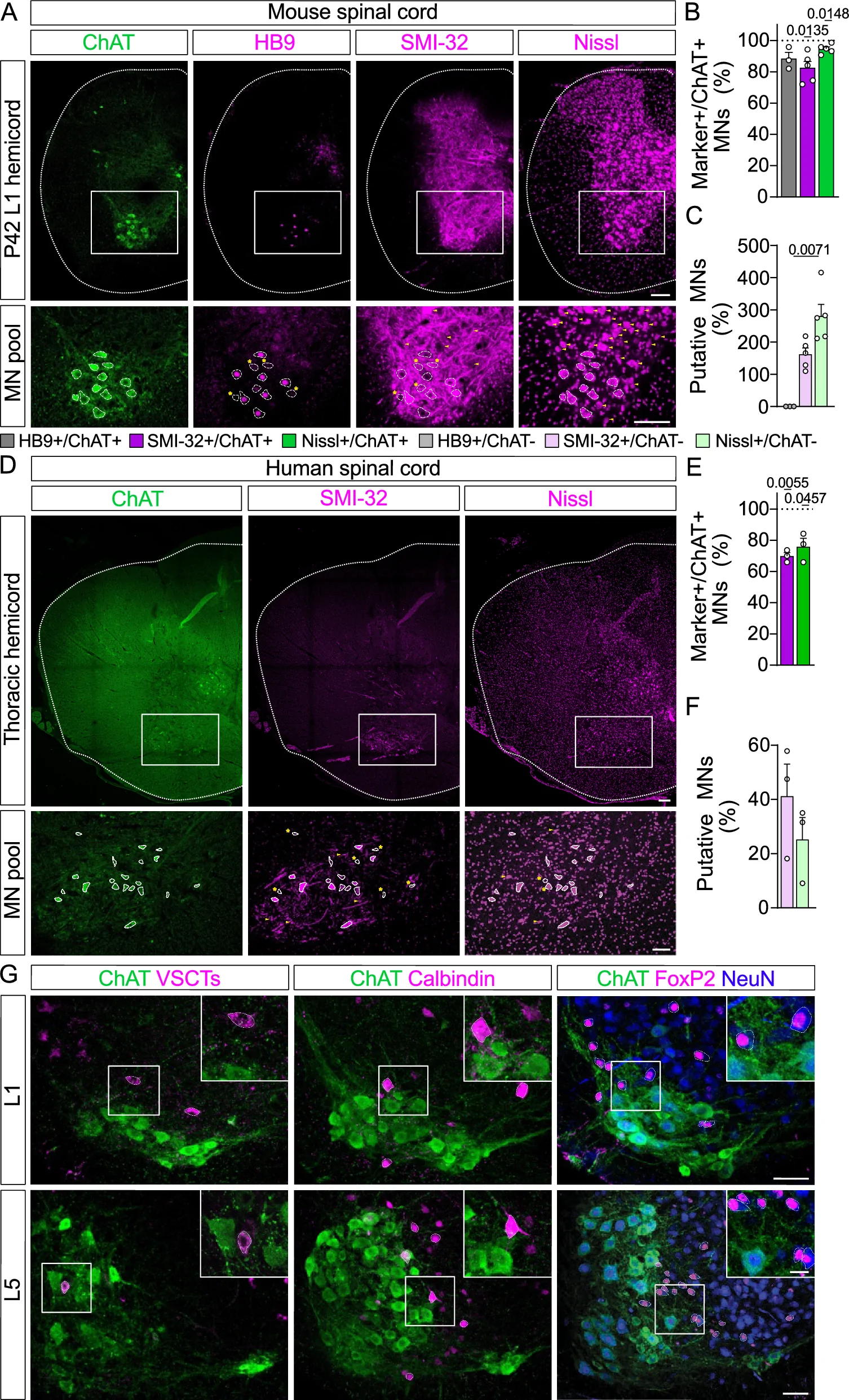
Specificity and off-target labeling of common motor neuron markers in mouse and human spinal cord. **A** Immunostaining of L1 hemicord (upper row) and MN pool (lower row) for ChAT (green), SMI-32, Nissl, and HB9 (magenta) from a P42 C57BL/6 mouse. Scale bars = 100 µm. Yellow asterisks indicate marker- ChAT+ MNs, and yellow arrows indicate marker+ ChAT- putative MNs. MN shapes, as identified by ChAT, are shown as white dotted outlines. **B**, **C** Quantification of SMI-32, Nissl, and HB9 labeling in ChAT+ MNs (B) and in ChAT- cells classified as putative MNs (C), expressed as percentages within the L1 ventral horn surrounding the MN pool of P42 C57BL/6 mice (n = 3 for HB9; n = 5 for SMI-32 and Nissl), as indicated by the white box in **A**. **D** Immunostaining of thoracic hemicord (upper row) and MN pool (lower row) for ChAT (green), SMI-32, and Nissl (magenta) from a 9-month-old human spinal cord sample. Scale bars = 200 µm (upper) and 100 µm (lower). Yellow asterisks indicate marker- ChAT+ MNs, and yellow arrows indicate marker+ ChAT- putative MNs. MN outlines identified by ChAT are shown as white dotted outlines. **E**, **F** Quantification of SMI-32 and Nissl labeling in human thoracic spinal cord samples in ChAT+ MNs **E** and in ChAT- cells (putative MNs) **F**, expressed as percentages within the ventral horn surrounding the MN pool (n = 3), as indicated by the white box in **D**. **G** Interneurons (magenta) in the ventral horn identified by CTB injection into the cerebellar vermis to label VSCTs, Calbindin for Renshaw cells, and FoxP2 for V1 interneurons, co-stained with ChAT (green) in L1 (upper row) and L5 (lower row) of P4 C57BL/6 mice. NeuN (blue) is co-labeled in the FoxP2 row to visualize neuronal somata. Insets show higher magnification of interneurons in close proximity to MNs. Scale bars = 50 µm and 20 µm for insets. Outlines of interneurons are shown as white dotted lines. Statistical analysis was performed using one-sample t-tests against ChAT+ MN values (**B**, **E**), Kruskal–Wallis test **C**, or unpaired t-test **F**

After validating the reliability of ChAT immunostaining in marking MNs, we assessed the specificity of other commonly used markers (Fig. 1A and Fig. S1C), using ventral roots as landmarks as defined by Approach 3 (Fig. 2A). We performed quadruple labeling with antibodies against ChAT, HB9, SMI-32, and Nissl on spinal cords from P42 C57BL/6 mice focusing on L1 and L5 segments that innervate proximal/axial and distal muscles, respectively [40]. HB9 and Nissl each labeled ~ 90–95% of all ChAT+ MNs, while SMI-32 labeled ~ 80% in both segments (Fig. 3A, B and Fig. S3A, B). Importantly, while HB9 selectively marked both α- and γ-MNs (Fig. S3G), SMI-32 and Nissl exhibited substantial off-target labeling in the ventral horn, detecting ~ 160% (SMI-32) and ~ 280% (Nissl) additional non-MNs – i.e. spinal interneurons – in L1 and ~ 75% and ~ 120% in L5 in mice, respectively (Fig. 3A, C and Fig. S3A, C). To assess whether soma size distinguishes true MNs from off-target profiles, we measured cross-sectional soma areas across marker combinations. ChAT+ MNs showed consistent soma sizes regardless of co-labeling, with mean areas of ~ 440–490 µm^2^ across the three markers (Fig. S3D, E). Notably, the soma size distribution of ChAT+ MNs appeared bimodal, consistent with previous reports describing distinct γ- and α-MN populations [45, 46]. In contrast, ChAT- profiles labeled by SMI-32 or Nissl had smaller mean soma areas (~ 275–300 µm^2^) and overlapped predominantly with the smaller ChAT+ population (Fig. S3D, F). These findings indicate that soma size alone cannot reliably distinguish true MNs from off-target profiles, particularly within the smaller end of the MN population.

Extending these findings from mice to human autopsy tissue, we analyzed 20 µm cryosections of postmortem thoracic spinal cord tissue. Nissl and SMI-32 labeling identified ~ 70–75% of ChAT+ MNs, while both markers exhibited substantially fewer off-target profiles in humans (~ 25–40%) compared to mice (Fig. 3D–F).

To identify large ChAT- neurons located within or adjacent to MN pools, we immunolabeled subsets of ventral interneurons, including ventral spinocerebellar tract (VSCT) neurons identified by injecting cholera toxin B subunit (CTB +) in the cerebellum, calbindin+ Renshaw cells, and FoxP2+ V1 interneurons in L1 and L5 spinal segments of P4 C57BL/6 mice [31, 47, 48]. Some interneurons were similar in size to small MNs, whereas others, such as VSCT neurons, were MN–sized and located adjacent to or within MN pools (Fig. 3G).

Next, we addressed whether different developmental stages and tissue fixation during processing could introduce variability in MN counts. To assess potential fixation effects on MN counts during maturation, we examined spinal cords from P10 and P60 ChAT-eGFP mice, rapidly dissected in ~ 4 °C oxygenated aCSF with subsequent immersion-fixation with 4% PFA. L1 segments, identified by ventral root landmarks, were sectioned at a vibratome. At P10, GFP-expressing MNs in immersion-fixed tissue revealed nearly complete colocalization with ChAT and HB9 and partial colocalization with SMI-32 (Fig. S3H). In contrast, immersion-fixed P60 spinal cords lacked detectable GFP, even with antibody enhancement or ChAT signals in MNs, whereas HB9 and SMI-32 remained visible (Fig. S3H). By comparison, transcardially perfused adult spinal cords exhibited robust ChAT staining (Fig. S3A, G), indicating that cytoplasmic ChAT degrades more rapidly than nuclear HB9 or cytoskeleton-enriched SMI-32 in immersion-fixed adult spinal cords, underscoring the necessity of perfusion for MN-specific labeling with ChAT in adult, fully myelinated tissue.

These findings identify specific interneurons as a likely source of MN misclassification when using nonspecific markers such as SMI-32 or Nissl, highlight the importance of perfusion for preserving ChAT signal, and demonstrate HB9 and ChAT as highly specific markers for accurate manual MN quantification.

### Deep learning–based segmentation enables unbiased motor neuron quantification within the intact spinal cord

**Fig. 4:**
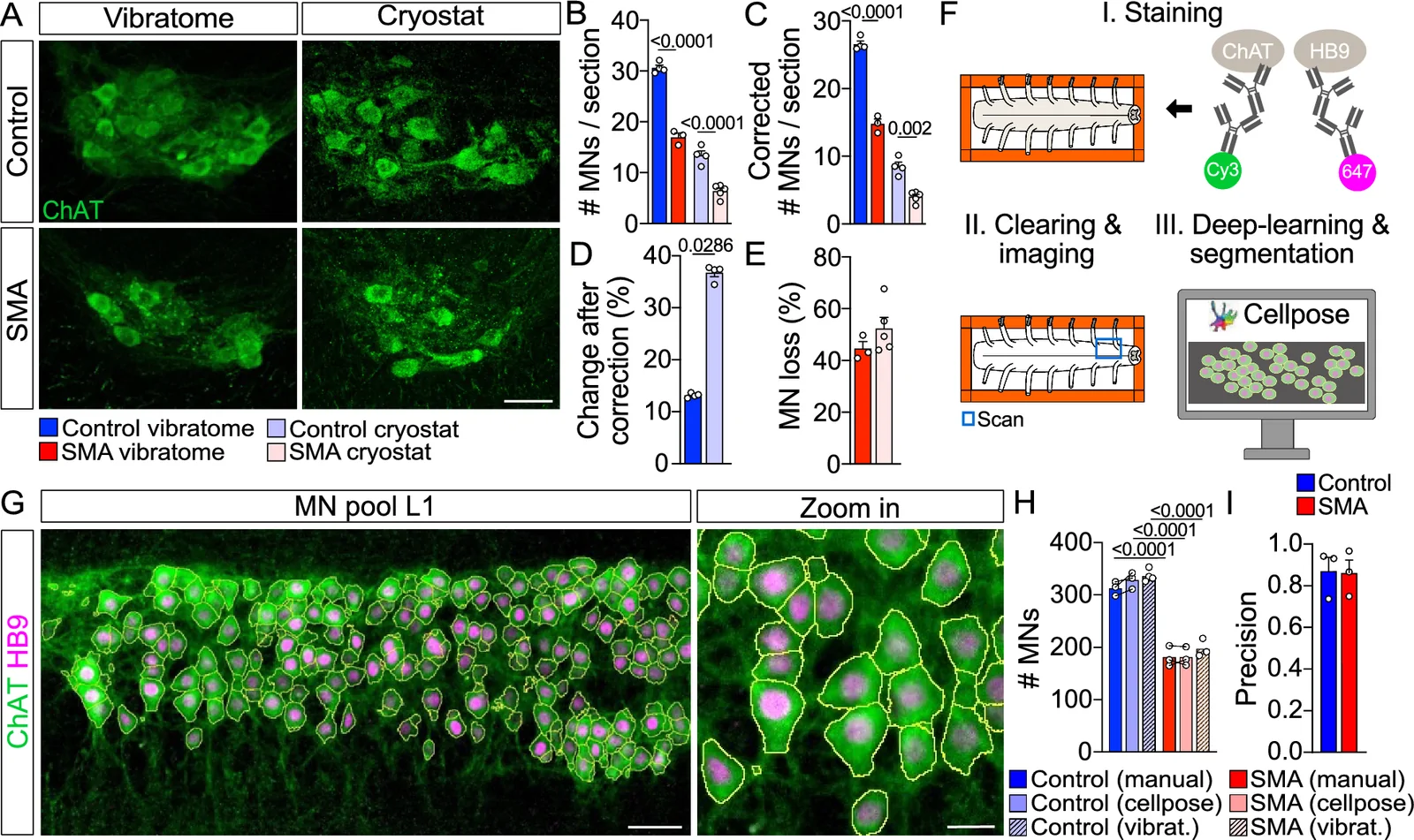
Deep learning–based Cellpose automated MN quantification confirms manual vibratome-based counts. **A** Immunostaining of the L1 MN pool for ChAT (green) in sections cut using a vibratome (75 µm) or cryostat (20 µm) from end-stage control and SMNΔ7 mice. Scale bar = 50 µm. **B** Quantification of MNs per section for the groups shown in **A** (for both sectioning methods, control: n = 4, SMN∆7 vibratome: n = 3, SMA cryostat: n = 5). **C** Quantification of Abercrombie-corrected MNs per section for the same groups as in **B**. **D** Percent change after Abercrombie correction for both cryostat and vibratome control groups. **E** Calculated MN loss (%) between SMN∆7 and control animals for the same groups as in **B** (vibratome: n = 3; cryostat: n = 5). **F** Schematic of the workflow for automated analysis using Cellpose of whole-mount, immunostained, cleared spinal cords. The blue box indicates the scanned area of the L1 segment. **G** Immunostaining of cleared whole-mount L1 MN pool for ChAT (green) and HB9 (magenta) from a C57BL/6 mouse at P4, segmented with Cellpose (yellow outlines). Scale bars = 50 µm and 20 µm. **H** Quantification of MNs in the entire L1 segment of P10 control and SMNΔ7 mice (n = 3) using manual and Cellpose-based segmentation, as well as from L1 segments cut using a vibratome (control: n = 4; SMA: n = 3). Connecting lines indicate measurements from the same animal for manual and Cellpose-based quantification. **I** Calculated precision of Cellpose MN quantification in the L1 segment of P10 control and SMNΔ7 mice (n = 3), compared to ground-truth manual quantification. TP = true positives, FP = false positives, FN = false negatives. Statistical analysis was performed using one-way ANOVA (**B**, **C**, **H**), Mann–Whitney test (**D**), and unpaired t-test (**E**, **I**)

In addition to tissue fixation and marker selection, variability in sectioning methods and manual MN quantification across investigators constitute potential sources of error. To evaluate these possibilities, we first examined how vibratome versus cryostat sectioning affects MN integrity and counts. We sectioned entire L1 spinal segments from perfused P10 FVB control mice of the SMNΔ7 line using both methods, followed by ChAT immunofluorescence. As expected, 75 µm vibratome sections consistently contained higher MN counts than 20 µm cryostat sections (Fig. 4A, B). To correct for overestimation due to nuclei split across adjacent sections, we measured the MN nuclear diameter (Fig. S4A) and applied the Abercrombie correction [49]. MN counts in thicker vibratome sections were reduced by only ~ 10%, whereas thin cryostat sections required ~ 35% adjustment (Fig. 4C, D). To test whether this variability persists under neurodegenerative conditions, we performed the same analysis in end-stage P10 SMNΔ7 mutant littermates [18]. Both methods reliably detected a ~ 50% reduction in L1 MNs per section in SMNΔ7 mice compared to controls, consistent with previous reports [26, 43, 50–53]. This loss is independent of the Abercrombie correction, as MN nuclear diameters are unaltered in SMA (Fig. 4A–E and Fig. S4A).

These findings indicate that both sectioning methods reliably detect MN loss in SMNΔ7 mice. However, because thinner sections require substantially larger profile-based corrections, section thickness may influence the accuracy of absolute MN estimates, even when relative disease-associated differences are preserved.

To directly compare vibratome section-based manual counts with the true MN population of the intact spinal cord, we applied a deep-learning, automated pipeline for unbiased, whole-tissue quantification (Fig. 4F). To do so, we dissected spinal cords of perfused P10 FVB control and SMNΔ7 mice and pinned them into a 3D printer custom-made chamber (Fig. S4B). Subsequently, we immunolabeled the dissected spinal cord for ChAT and HB9, cleared the tissue, and imaged the entire L1 segment identified using ventral root landmarks. The confocal stacks were uploaded into the open-source, deep-learning software Cellpose [54]. Representative images of these stacks were used to train an existing Cellpose model [55] to segment and quantify MNs based on ChAT signal with additional help of HB9 nuclear labeling (Fig. 4F, G and Fig. S4C). The trained Cellpose model generated MN counts that closely matched manual assessments of the same stacks and parallel vibratome L1 sections without Abercrombie correction, accurately reflecting the degree of MN loss (Fig. 4H). Quantification of MNs with Cellpose achieved a precision of 0.86, indicating that the majority of automatically detected MNs corresponded to manually identified MNs with few false-positive detections in both control and SMA mice (Fig. 4I). This automated approach establishes an unbiased method for reliable MN quantification of intact spinal cord tissue and validates the manual vibratome sectional analysis under both healthy and disease conditions.

### Random sampling masks spinal column-specific variability of MN loss in SMA

**Fig. 5:**
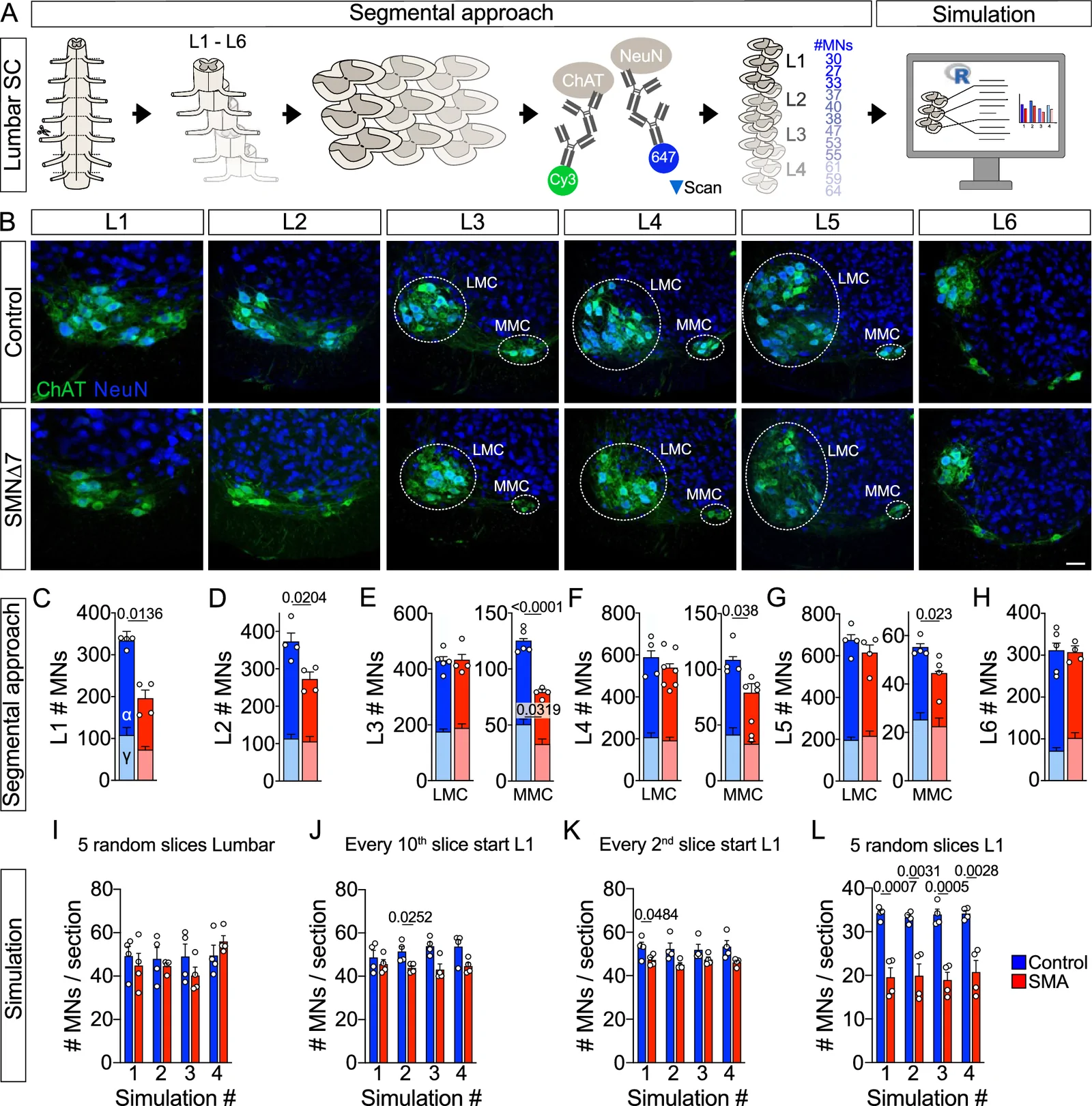
Non-segmented counting approaches mask selective MN vulnerability in severe SMA mice. **A** Schematic of the workflow for manual segment-specific MN quantification in lumbar spinal cords, followed by simulation of randomized segmental sectioning implemented in R. **B** Immunostaining of L1–L6 spinal segments for ChAT (green) and NeuN (blue) from P10 control (upper row) and SMNΔ7 (lower row) mice. ChAT+/NeuN− = γ-MNs; ChAT+/NeuN+ = α-MNs. Scale bar = 50 µm. LMC and MMC are outlined in L3–L5 with white dotted lines. **C**–**H** Quantification of MN numbers in L1 **C**, L2 **D**, L3 **E**, L4 **F**, L5 **G**, and L6 **H** spinal segments of P10 control and SMNΔ7 mice (L1: n = 4 per group; L2: n = 4 per group; L3: control n = 5, SMNΔ7 n = 4; L4: control n = 4, SMNΔ7 n = 7; L5: n = 4 per group; L6: control n = 6, SMNΔ7 n = 4). Segments L3–L5 are further subdivided into LMC and MMC. **I**–**L** Results of randomization simulations of MN number per slice in control versus SMN∆7 mice under different sampling conditions: **I** five random slices from L1–L6 (lumbar); **J** every 10th slice after a random start within L1; **K** every 2nd slice after a random start within L1; and **L** five random slices from L1 (n = 4 per group). Four simulation rounds are shown (1–4). Statistical analysis was performed using unpaired t-test (**C**–**H**, **I**–**L**), Welch’s t-test (F, MMC α-MNs), and Mann–Whitney test (H α-MNs; I, round 2; J, rounds 3–4; K, rounds 2–3)

Our meta-analysis identified inconsistent segment sampling as a key variable in reported MN loss across SMA studies (Fig. 1). With a validated ChAT-based sectional approach in place, we next examined the consequences of random sampling by quantifying MN loss across the entire lumbar spinal cord of control and end-stage (P10) SMN∆7 mice by integrating these data into a computational model (Fig. 5A). We applied ChAT and NeuN immunolabeling on 75 µm thick L1 to L6 vibratome sections to distinguish α-MNs innervating extrafusal muscle fibers (ChAT+, NeuN +) from γ-MNs innervating intrafusal fibers (ChAT+, NeuN-) [45, 46]. Whereas L1–L2 and MMC MNs across L3–L5 innervate proximal and axial muscles, LMC MNs and dispersed L6 pools innervate distal hindlimb muscles [40]. γ-MNs were spared in all distal muscle–innervating pools with trends for modest reductions observed in proximal muscle–innervating pools. Degeneration remained restricted to α-MNs innervating proximal and axial muscles throughout the lumbar spinal cord with severity decreasing from upper to lower lumbar segments (Fig. 5B–H).

Next, we incorporated these newly acquired ChAT+ MN counts across the entire lumbar spinal cord into an R-based modeling script (Fig. 5A and I–L, Fig. S5) to simulate random section-subsampling strategies used in previous MN quantification studies [56–58]. Four rounds of simulated random sampling of five consecutive lumbar sections failed to detect significant MN loss between control and SMN∆7 animals (Fig. 5I). Similarly, sampling every 2nd or 10th section throughout the lumbar spinal cord, starting at a random point in L1, produced a significant difference in only one instance, with a minor effect size (Fig. 5J, K). In contrast, sampling five random sections from L1 or L2 consistently detected significant MN loss in all four simulation rounds (Fig. 5L and Fig. S5).

These findings highlight that random sampling across lumbar spinal cord segments carries a high risk of misestimating MN loss in SMA due to segment- and pool-specific heterogeneity in degeneration.

### Column-specific MN vulnerability differs among mouse models of MN disease

**Fig. 6:**
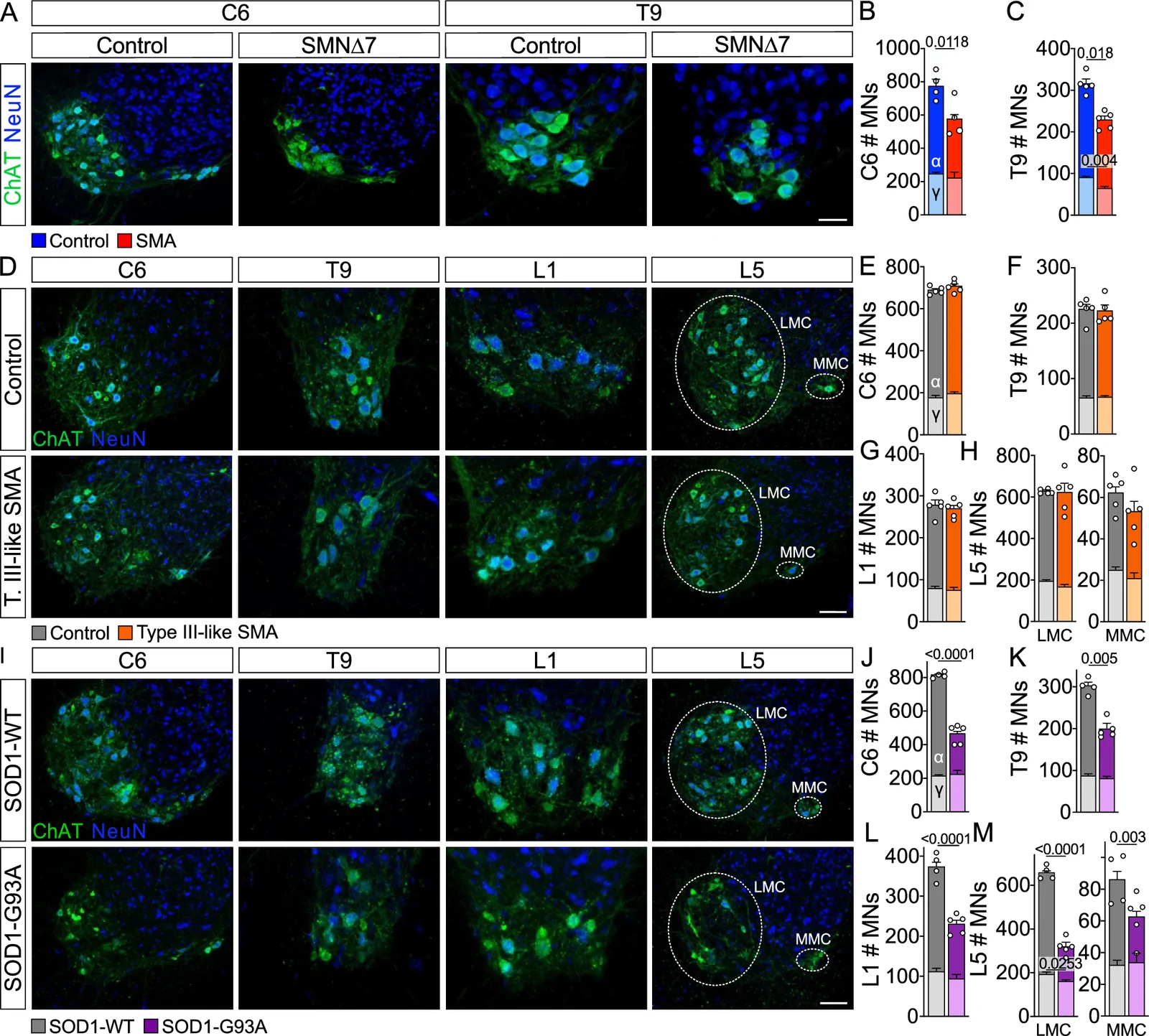
Differential vulnerability of MN pools across mouse models of motor neuron disease. **A** Immunostaining of C6 and T9 spinal segments for ChAT (green) and NeuN (blue) in P10 control (upper row) and SMNΔ7 (lower row) mice. ChAT+/NeuN− cells correspond to γ-MNs, whereas ChAT+/NeuN+ cells correspond to α-MNs. Scale bar = 100 µm in C6 and 40 µm in T9. **B**, **C** Quantification of MN pools in C6 **B** and T9 **C** segments of P10 control and SMNΔ7 mice (C6: n = 4 per group; T9: n = 5 per group). **D** Immunostaining of C6, T9, L1, and L5 spinal segments for ChAT (green) and NeuN (blue) in P270 control (upper row) and Type III-like SMA (lower row) mice. Scale bar = 100 µm in C6 and L5, and 50 µm in L1 and T9. In L5, the LMC and MMC are outlined with white dotted lines. **E**–**H** Quantification of MN pools in C6 **E**, T9 **F**, L1 **G**, and L5 LMC and MMC (H) segments of P270 control and Type III-like SMA mice (n = 5 per group). **I** Immunostaining of C6, T9, L1, and L5 spinal segments for ChAT (green) and NeuN (blue) in P130 SOD1-WT (upper row) and SOD1-G93A (lower row) mice. Scale bar = 100 µm in C6 and L5, and 50 µm in L1 and T9. In L5, the LMC and MMC are outlined with white dotted lines. **J**–**M** Quantification of MN pools in C6 **J**, T9 **K**, L1 **L**, and L5 LMC and MMC **M** segments of P130 SOD1-WT and SOD1-G93A mice (control: n = 4; ALS: n = 5). Statistical analysis was performed using an unpaired t-test **B**, **C**, **E**–**H**, **J**–**M**, a Mann–Whitney test (F α-MNs, H LMC γ-MNs, K γ-MNs), or Welch’s t-test (G γ-MNs, H LMC α-MNs)

Our meta-analysis (Fig. 1) and random sampling (Fig. 5) suggested that the selective vulnerability of MN pools innervating proximal/axial muscles accounts for lumbar MN loss in severe SMN∆7 mice. To determine whether this pattern extends beyond the lumbar cord, we applied ChAT and NeuN immunostaining to perfused end-stage P10 SMN∆7 mice to quantify MN loss in cervical (C6) and thoracic (T9) segments which primarily innervate proximal forelimb and abdominal/trunk muscles, respectively (Fig. 6A). In accord with our observation within the lumbar spinal cord, C6 and T9 MN pools exhibited a significant (~ 30%) α-MN loss and an additional ~ 30% reduction in γ-MNs in T9 (Fig. 6A–C). Consistent with a previous report in the same mouse model [26], neither γ-MNs nor α-MNs exhibited significantly reduced soma size in SMN∆7 mice (Fig. S6A–D). In contrast to these observations in severe SMA mice, human autopsy spinal cords from SMA type I patients showed a ~ 50% reduction in α-MN soma size, whereas γ-MNs showed a ~ 25% reduction that did not reach statistical significance (Fig. S6E–H). Together, these data reveal a consistent, strong loss of α-MNs innervating proximal and axial muscles and modest vulnerability of γ-MNs at distinct spinal cord levels in severe SMN∆7 mice.

Next, we tested whether the selective vulnerability of α-MNs innervating proximal muscles is conserved in an adult-onset, 4-copy SMN2 Type III-like SMA mouse model, which has been reported to exhibit ~ 30% lumbar MN loss at P270 on a C57BL/6 background based on analyses with non-specific MN markers [59]. To investigate this, we analyzed both α- and γ-MNs using ChAT and NeuN in C6, T9, L1, and L5 segments isolated from perfused Type III-like SMA mutant mice and age-matched controls at P180 and P270. Strikingly, we found that α- and γ-MN numbers were fully preserved across all segments and ages (Fig. 6D–H and Fig. S6I–L). Consistent with this, p53 pathway activation, which drives MN death in severe and intermediate SMA mouse models [20, 43, 51, 60], was completely absent in the nuclei of L1 MNs of Type III-like SMA mice (Fig. S6M). Accordingly, neuromuscular junctions (NMJs) in the distal tibialis anterior and axial quadratus lumborum muscles were fully innervated (Fig. S6N–P), unlike in severe and intermediate SMA mouse models [20]. Together, these findings demonstrate the absence of MN loss in the 4-copy SMN2 Type III-like mouse model of SMA.

Finally, we investigated segment-specific MN loss in another adult-onset MN disease, amyotrophic lateral sclerosis (ALS), using the established SOD1-G93A mouse model [28]. We analyzed MNs in the C6, T9, L1, and L5 segments at end-stage (P130) in SOD1-G93A mutants and SOD1-WT controls. In SOD1-G93A mutants, α-MNs exhibited substantial degeneration (> 50%) across all segments, with the distal muscle–innervating L5 LMC MNs showing the greatest loss (Fig. 6I–M), consistent with the distal-onset phenotype characteristic of ALS rodent models [11, 27, 28]. We also found that α-MNs were significantly reduced in size in this mouse model (Fig. S6Q–S). In contrast, γ-MNs remained largely unaffected in numbers and size (Fig. S6T), in accordance with previous reports [61, 62].

Taken together, segment-specific analyses highlight the importance of specific markers in revealing extensive α-MN loss in SOD1-G93A mice, selective proximal vulnerability in severe SMNΔ7 mice, and the absence of MN degeneration in the Type III–like SMA model.

## Discussion

MN degeneration is a hallmark of several neurodegenerative disorders, yet reproducible quantification of MN loss remains technically challenging, limiting accurate disease phenotyping and assessment of therapeutic efficacy in preclinical animal studies. We identified substantial variability in reported MN loss across studies using identical SMA mouse models, for which spinal cord segment sampling emerged as a major contributor with additional contributions from tissue processing and MN marker selection. To overcome these limitations, we demonstrated the superior accuracy of a segment-specific, ChAT-based manual sectioning strategy, validated using unbiased, automated machine learning–based whole-mount counting. Applied to SMA and ALS mouse models, this approach revealed disease-specific spatial selective MN vulnerability, demonstrating that methodological rather than biological differences underlie previously reported variability and provided a framework to enhance reproducibility across studies.

Our SLR identified > 60% of variability in MN loss across the three most commonly used SMA mouse models. Given that MN death is a defining hallmark of MN diseases, such variability in this key readout may substantially bias mechanistic interpretation and the evaluation of therapeutic efficacy. Our meta-analysis suggested that the examined spinal region is a major source of variability in reported MN loss and that segment–specific upper lumbar and medial MN pools innervating axial/proximal muscles are consistently more affected than lateral MNs innervating distal muscles in SMNΔ7 mice, reflecting the proximo-distal gradient of weakness in SMA patients [25, 26]. However, methodological differences likely contribute additional heterogeneity across studies. Because spinal segment selection emerged as the most consistent variable, we directly tested this anatomical selectivity by validating a sectional ChAT-based quantification method [20, 26, 43] within intact, cleared spinal cords using high-resolution confocal microscopy.

This sectional approach yielded precise, segment-specific MN numbers across neonatal and adult mice, establishing a reliable methodology for standardized, cross-study comparisons. The implementation of an automated deep-learning workflow using Cellpose [54, 55] for reliable MN quantification of intact spinal cords offered an unbiased alternative and further validated this sectional approach. Together, these validation approaches establish segment-specific ChAT-based analysis of vibratome sections as a practical, reliable, and reproducible method for routine MN quantification.

Using MN counts acquired from applying this sectional approach to the entire lumbar spinal cord in SMNΔ7 mice, we were able to confirm the enhanced vulnerability of proximal muscle-innervating MN pools established in our meta-analysis. Moreover, computational simulations of randomly selected spinal segments in control and SMN∆7 mice identified inconsistent rostrocaudal sampling of MN populations as a major source of interstudy variability, demonstrating that non-stratified section sampling across anatomically heterogeneous lumbar regions can obscure selective MN vulnerability patterns. [39, 63, 64]. In contrast, restricting these analyses to MN pools that innervate proximal muscles produced robust, reproducible patterns of degeneration. Together, our established anatomically standardized manual and automated quantification methods provided a platform for MN quantification in health and disease and identified inconsistent spinal segment selection as a major driver of variability in MN numbers.

Although nonspecific spinal segment sampling may help explain most of the observed interstudy variability, additional methodological factors such as spinal cord processing and selection of appropriate marker(s) can affect MN quantification beyond anatomical sampling. Spinal cord extraction by corpectomy preserves segmental integrity and enables reliable segment identification using vertebral or ventral root landmarks [53, 65]. In contrast, hydraulic extrusion damages spinal roots and requires immersion fixation, which compromises immunoreactivity [63, 66], particularly in adult mice, as we have shown here. While the sectioning method had minimal impact on the reliability of MN quantification and observed loss in SMN∆7 mice, thicker vibratome sections reduced double-counting and artifacts, especially since the rostro-caudal length of spinal segments changes during development or pathology [20]. However, counting accuracy is influenced not only by section thickness but also by counting rules, correction methods, and the choice of counting reference. Nevertheless, the close agreement between vibratome-based and whole-mount counts supports the utility of this approach for routine MN quantification.

Beyond sampling and tissue processing, the choice of MN marker influences the accuracy of MN quantification. Retrograde tracer injections and ventral root labeling provide muscle-specific or segment-specific MN identification but are invasive and may become unreliable when axonal degeneration or impaired axonal transport precedes MN loss, while their technical demands and developmental constraints limit their applicability in disease mouse models with short survival windows [26, 67, 68]. In our study, nearly all retrogradely labeled neurons expressed ChAT, highlighting the importance of combining retrograde labeling with ChAT immunostaining to ensure specific MN identification. However, inclusion of ChAT+ spinal neurons in the intermediate zone [38], together with potential downregulation of ChAT expression under neurodegenerative conditions—although no studies have reported to-date any significant reduction in ChAT immunoreactivity under neurodegenerative diseases—may require a different motor neuron marker, such as Hb9. Similar to ChAT, HB9 labeled ~ 90% of α- and γ-MNs with minimal off-target labeling as previously described [46, 69]. Whereas SMI-32 is largely confined to proximal neurites [32, 33] and under-detects MNs, Nissl staining marks nearly all MNs. However, both SMI-32 and Nissl staining also label a large number of non-MNs in the ventral horn, namely spinal interneurons. Our data indicate that these include ventral spinocerebellar tract neurons, Renshaw cells, and FoxP2+ neurons. Importantly, these ChAT- neuronal populations predominantly overlapped with the lower end of the ChAT+ MN soma-size distribution, where γ-MNs and smaller α-MNs reside. Thus, even when soma size, morphology, and anatomical location are considered, nonspecific markers may result in preferential misclassification of smaller MN populations. It is likely that additional ventral interneuron populations, such as V0, V2a/V2b, and V3 cells, may also be misassigned as MNs using SMI-32 and Nissl staining [70]. Avoiding the possible inclusion of interneurons into MN counts should be taken into great consideration as interneuron vulnerability could vary from MNs and confound results in MN disease mouse models. Within certain disease models, selective shrinkage of MN soma size further exacerbates this limitation [62, 71], rendering size- or location-based classification with nonspecific markers unreliable. Notably, mislabeling of non-MNs is a less prominent issue in human than murine spinal cords, likely due to the larger size and reduced density of MNs [72]. Although conventional histological approaches remain valuable for routine neuropathological assessment and morphological characterization of degenerating neurons, our results demonstrate that rigorous spinal cord processing and the use of selective MN markers are essential for specific and reliable MN quantification in mice.

Having established a highly reliable MN quantification method in wild-type mice, we applied this approach to reassess regional vulnerability across the spinal cord in the most widely studied mouse models of ALS and SMA [18, 28]. At end-stage in SOD1-G93A mice, α-MN degeneration was uniform across all spinal motor pools, whereas SMNΔ7 mice exhibited selective vulnerability restricted to MNs innervating axial and proximal muscles. These observations suggest that, in SMA more so than in ALS, a substantial subset of MNs is intrinsically resistant to neurodegeneration and identifies selective motor pool involvement as a major biological contributor to the variability reported across SMA studies. Consistent with this notion, autopsy studies of SMA Type I patients have repeatedly reported that ~ 50% of MNs are preserved [73–76]. Although α-MNs are well established as the primary vulnerable population, γ-MNs have generally been considered resistant in both SOD1-G93A and SMA models [61, 62, 71]. In concordance, we detected no γ-MN loss in SOD1-G93A mice. Interestingly, spinal segment-specific analysis in SMNΔ7 mice revealed a modest reduction in a subset of γ-MN pools innervating proximal and axial muscles, suggesting that they are not entirely spared but substantially less vulnerable than α-MNs. The extent of this pathology may be underestimated due to the short lifespan of this severe SMA model. Consistent with this interpretation, analyses of SMA patient tissue report γ-MN reductions comparable to α-MN loss [75]. In contrast to the selective MN loss observed in SMNΔ7 mice, we detected no MN degeneration across the spinal cord in 9-month-old Type III–like SMA mice on a C57BL/6 background. This differs from a previous report describing > 30% late-onset loss of MNs innervating distal muscles [59], although these MNs are consistently spared across SMA mouse models [20, 26]. Consistent with the absence of MN loss, our study demonstrates that NMJs of both axial and distal muscles remained fully innervated in Type III–like SMA mice, consistent with previously reported preservation of muscle function at this age [59]. The use of non-specific MN markers may explain conflicting reports from studies of this model on an FVB background, which range from early, non-progressive MN loss to a gradual ~ 30% decline by one year of age [77, 78]. Importantly, the severe Taiwanese SMA model, generated by breeding Type III–like SMA mice yet carrying fewer SMN copies, shows no MN loss across both C57BL/6 and FVB backgrounds when assessed using the same validated sectional quantification [20, 26], indicating that these discrepancies do not stem from genetic backgrounds. Two independent reports using the same approach also demonstrated the absence of MN loss in the milder end-stage Smn^2B/–^ model, although minor, selective MN degeneration emerges beyond median survival [20, 79]. Consistent with this, convergent upregulation and phosphorylation of p53 in MNs drives their degeneration in SMNΔ7 and Smn^2B/−^ mice [20, 43, 51, 60], whereas this response is absent in Taiwanese [20] and Type III-like SMA mice, linking cell-autonomous p53 activation to MN degeneration across SMA models. Accordingly, the > 60% variability in reported MN loss across SMA mouse models identified in our SLR and meta-analysis is likely driven primarily by methodological differences, with robust MN degeneration most consistently observed in the SMNΔ7 model.

In conclusion, our study establishes a standardized, validated, and scalable framework for MN identification and quantification in mouse models across normal development and in the context of injury and neurodegeneration. By reducing methodological variability and enabling reproducible analyses, this approach advances the rigor and translational significance of preclinical studies, ultimately facilitating the development and evaluation of therapeutic strategies for MN disorders.

## Materials and methods

### Systematic literature review

SMA spinal cord studies reporting MN loss between untreated mutant and control littermates were included in the SLR. All relevant studies up to February 23, 2023 were identified in peer-reviewed journals indexed in PubMed using a search strategy described in Table S1.

Identified studies were included if they met the pre-specified PICOS criteria adapted for animal studies (population, intervention, comparator, outcomes, and study types; see Table S2). To be included in the SLR, studies (n = 75) had to utilize at least one of three predefined SMA mouse models (SMN∆7, Smn^2B/–^, or Taiwanese), identified based on breeding origin or genetic model of the mouse; the experimental pairs (mutant and control) had to include at least n = 3; and animals had to be sacrificed within a predetermined, model specific end-stage timeframe, capped by a model-specific maximum postnatal day. MN counts had to be obtained ex vivo from the ventral region of the mouse spinal cord. Any mice receiving treatment were excluded from MN counts. MN count means for study cohorts were extracted from publication text or reliably estimated using the open-source software WebPlotDigitizer [80]. Exact details for each of the screening criteria are included in Table S3.

### Meta-analysis

A subset of publications meeting the SLR criteria for the SMNΔ7 model was included in the meta-analysis (43 of 75; Table S3). Pooled effect sizes, a metric demonstrating the magnitude and direction of a relationship between two variables, were acquired by calculating Cohen’s d using random-effects models based on between-group standardized mean differences, the type of effect size matching our model, and corrected for small sample sizes (n < 21) with Hedges’ g. MN count means were pooled and their confidence intervals were obtained either via WebPlotDigitizer or from the figure summaries within the original publications. For studies reporting standard deviations (SD), 95% confidence intervals were calculated from the SDs and corresponding sample sizes. Heterogeneity across studies was quantified via the I^2^ statistic, which estimates the proportion of total variability in effect estimates attributable to between-study heterogeneity rather than sampling error. Higher I^2^ values indicate greater between-study variability, suggesting that differences in study-level characteristics may contribute to the observed outcomes. By contrast, lower I^2^ values indicate more homogeneity across studies, implying that the observed variability is more likely due to chance. A feature of I^2^ is its relative independence from the number of studies included within a meta-analysis. I^2^ is commonly expressed as a percentage and interpreted using conventional thresholds: 0% (none), 25% (low), 50% (medium) and 75% (high) [81].

### Subgroup analysis and multilevel analysis

The reported heterogeneity of the meta-analysis was further explored using subgroup or moderator analyses to examine potential sources of between-study heterogeneity. This is done under the premise that not all studies originate from the same population and may differ in their underlying true effects due to study-level characteristics. Groupings of experiments according to hypothesized effect modifiers were tested using a fixed-effects model to assess for differences between subgroups. Within each subgroup, a random-effects model was utilized and a test for heterogeneity was performed. The effects for each subgroup were pooled and compared across subgroups. A change in the I^2^ value was examined descriptively to assess whether the hypothesized variable may contribute to the variation observed between studies. To prevent skewing data towards authors with multiple experiments included in the meta-analysis, multilevel analyses were conducted to account for the non-independence of effect sizes**.** The R statistical software (Version 4.4.3, R Foundation for Statistical Computing, Vienna, Austria) was utilized for the meta-analysis. The R code and datasets required to replicate meta-analysis findings are publicly available on GitHub at https://github.com/a-l-norman/SMN-delta-7-meta-analysis-R-code.git using version v1.0.

### Mouse lines and genotyping

Breeding and experimental procedures were conducted in the animal facilities of Leipzig University (Leipzig, Saxony, Germany) and Columbia University (New York, NY, USA). All animal work at Leipzig University complied with European regulations (Council Directive 86/609/EEC), the German Animal Welfare Act (Tierschutzgesetz), and was approved by the regional authority (Landesdirektion Leipzig). Experiments at Columbia University were performed in accordance with the U.S. National Institutes of Health Guidelines for the Care and Use of Laboratory Animals and approved by the Columbia University Institutional Animal Care and Use Committee (IACUC). This included a 12 h/12 h light/dark cycle with unrestricted access to both food and water. The SMN∆7 mouse model (Smn±; SMN2+/+; SMNΔ7+/ +) on FVB background was obtained from Jackson Laboratory (stock #005025). The 4-copy SMN2 Type III-like SMA model was originally purchased on a FVB background from the Jackson Laboratory (stock # 005058, FVB.Cg-Tg(SMN2)2HungSmn1tm1Hung/J). Mice were then backcrossed over seven generations to a C57BL/6N background [82]. For ALS mice, the SOD1-WT B6SJL (JAX stock #002297) and SOD1-G93A B6SJL (JAX stock #002726) lines were used. ChAT-eGFP mice were obtained from the Jackson Laboratory (stock #007902). The following primers were used for genotyping: SMNΔ7: forward sequence (5′ to 3′) = GATGATTCTGACATTTGGGATG, reverse sequences (5′ to 3′) = TGGCTTATCTGGAGTTTCACAA and GAGTAACAACCCGTCGGATTC (wild-type band: 325 bp, mSmn ko: 411 bp). 4-copy SMN2 Type III-like SMA: forward sequences (5′ to 3′) = ATAACACCACCACTCTTACTC and GTAGCCGTGATGCCATTGTCA, reverse sequence (5′ to 3′) = AGCCTGAAGAACGAGATCAGC (wild-type band: 1050 bp, mSmn ko: 950 bp). SOD1-WT and SOD1-G93A: forward sequence (5’ to 3’) = CATCAGCCCTAATCCATCTGA, reverse sequence (5’ to 3’) = CGCGACTAACAATCAAAGTGA (transgene = 236 bp). ChAT-eGFP: forward sequence (5’ to 3’) = AAGTTCATCTGCACCACCG, reverse sequence (5’ to 3’) = CGTCGCCGATGGGGGTGTTC (transgene = 400 bp). For the SMN∆7 mouse line, control mice were obtained from the same litters as mutant mice. For the 4-copy SMN2 Type III-like SMA line, age-matched C57BL/6N mice were used as controls. For SOD1-G93A mice, age matching controls from the SOD1-WT line were taken. Experimental mice were monitored for welfare in accordance with approved animal protocol guidelines. Similar numbers of male and female mice were included, and data were combined, as no sex-specific differences in MN counts were observed or have been previously reported in SMN∆7, SOD1-G93A or 4-copy SMN2 Type III-like SMA mice.

### Immunofluorescence of sectioned murine and human spinal cord and murine muscles

Mice were transcardially perfused with phosphate-buffered saline (PBS) followed by 4% paraformaldehyde (PFA), and spinal cords were postfixed overnight in 4% PFA at 4 °C. For preparation of non-perfused immersion-fixed spinal cord segments from P10 and P60 ChAT-eGFP mice or end-stage SMN∆7 mice, spinal cords were dissected after animal-welfare-compliant decapitation into ice-cold, oxygenated (95%O_2_–5%CO_2_), and continuously perfused with artificial cerebrospinal fluid (aCSF) containing 113 mM NaCl, 3 mM KCl, 1 mM NaH_2_PO_4_.H_2_O, 25 mM NaHCO_3_, 11 mM D-glucose, 2 mM CaCl_2_.H_2_O and 2 mM MgSO_4_·7H_2_O. After dissection, spinal cords were immediately transferred to 4% PFA for fixation. Fixed tissue was stored in PBS containing 0.01% sodium azide (NaN_3_) until further processing.

Thoracic spinal cord from patients with SMA and controls were collected at Johns Hopkins University School of Medicine, Baltimore, MD, USA, as follows: controls: CNTL 12_04 (cause of death unknown), CNTL 12_06 (transverse myelitis; collected and analyzed tissue was not affected by transverse myelitis.), CNTL 13_01 (cardiac arrest) and CNTL 17_01 (pneumonia); and as SMA patients: SMA 10_01, SMA 14_04, SMA 14_05, SMA 17_04 and SMA 19_01. Expedited autopsies were conducted under parental- or patient-informed consent in strict observance of the legal and institutional ethical regulations. Tissues from patients with SMA and age-matched controls with a short post-mortem interval were included. All patient and control tissues used in this study are listed in Table S4.

Following spinal cord dissection via anterior corpectomy, mouse spinal cord segments were localized by their ventral roots as described previously [44]. For vibratome sectioning, individual segments were embedded in 5% agarose and serially sectioned transversely at 75 µm using a Leica VT1000S vibratome. For cryostat sectioning of human and mouse spinal cord tissue, segments were cryoprotected sequentially in 15% and 30% sucrose solutions at 4 °C for at least 2 h and overnight, respectively. The tissue was then embedded in Sakura Tissue-Tek O.C.T. compound and frozen in 2-methylbutane cooled with liquid nitrogen. Serial transverse Sects. (20 µm) were cut at − 20 °C on a Leica CM3050 S cryostat and stored at − 80 °C until use.

Human spinal cord sections were incubated for 20 min in Polyscience L.A.B. solution for antigen retrieval at room temperature and subsequently washed three times in PBS. Human and mouse spinal cord cryostat sections were blocked for 60 min at room temperature in PBS containing 5% normal donkey serum and 0.3% Triton X-100 (PBS-T, pH 7.4) and subsequently incubated with primary antibodies for a minimum of 18 h at 4 °C (see Table S5 for details). SMI-32 antibody was directly conjugated to Alexa 647 and did not require a secondary antibody for visualization. After incubation, tissues were washed three times for 10 min each in PBS, then incubated with appropriate fluorophore-conjugated secondary antibodies (Alexa 488, Cy3, Alexa 647, DyLight 405; Jackson ImmunoResearch) diluted 1:1000 in PBS-T for 3 h at room temperature. Following secondary antibody staining, sections were washed three times for 10 min in PBS, mounted on slides, covered with glass coverslips, and protected with an anti-fading solution of glycerol:PBS (3:7), as described previously [75].

Mouse spinal cord sections cut with a vibratome were blocked for 90 min at room temperature in 5% normal donkey serum (NDS) diluted in PBS-T and incubated overnight with primary antibodies diluted in blocking solution (see Table S5 for details). Afterwards, the sections were washed six times for 10 min each in PBS, then incubated with secondary antibodies diluted 1:1000 in PBS-T for 3 h at room temperature. Finally, sections were washed six times for 10 min in PBS, mounted on slides, covered with glass coverslips, and protected with a solution containing glycerol:PBS (3:7), as described previously [20]. Labeling of ventral spinocerebellar tract neurons was achieved via injection to the cerebellum of 1% cholera toxin B subunit (CTB) conjugated to Alexa 488 (Invitrogen) in P0 C57BL/6 mice, following established protocols [31].

For immunostaining of neuromuscular junctions (NMJs), desired muscles were dissected immediately after perfusion and post-fixed with 4% PFA overnight. After fixation, single muscle fibers were teased apart and washed three times in PBS for 10 min each, followed by incubation for 1 h at room temperature with a blocking solution consisting of PBS-T (0.3%) with 5% NDS. Afterwards mouse anti-neurofilament (NF) and anti-synaptic vesicle 2 (SV2) antibodies to immunolabel the presynaptic aspect of the NMJ were applied in blocking solution overnight at room temperature (Table S5). The muscle fibers were then washed three times for 10 min in PBS, followed by incubation with secondary antibodies and α-bungarotoxin (BTX) Alexa Fluor 555 diluted in PBS-T for 3 h at room temperature as described previously [83]. Lastly, muscle fibers were washed three times in PBS for 10 min and mounted on slides covered with glycerol:PBS (3:7).

### Retrograde filling, immunofluorescence and clearing of whole-mount spinal cord

Segment-specific retrograde filling of MNs via ventral roots was performed as established previously [26]. Briefly, the spinal cord of C57BL/6 P4 mice was dissected free under in vitro conditions in cooled (10 °C) and continuously perfused artificial CSF (aCSF). The appropriate ventral roots were inserted into suction electrodes and incubated in an alternating manner with either Texas Red Dextran or Fluorescein Dextran [10,000 molecular weight (MW)] for 24 h. Following this period, the lumbar spinal cord was immersion-fixed in 4% PFA for 24 h. Spinal cords that were processed for analysis with Cellpose software, were pinned into a custom-made silicone chamber using a 3D-printed mold designed and produced by Juergen Simon, Steffen, and Ingo Rueger. Afterwards, lumbar spinal cords were processed for whole-mount immunostaining and subsequent clearing. All steps were performed in light-protected conditions, on an orbital shaker, in 2 ml Eppendorf microtubes and with solutions containing 0.02% NaN_3_. Spinal cords were permeabilized for 48 h in PBS with 2% Triton X-100 (2% PBS-T) at room temperature and then blocked for 48 h at 4 °C in 1% PBS-T supplemented with 2.5% DMSO and 10% NDS. Next, they were incubated for 4 days at 4 °C with ChAT antibody (Millipore) diluted 1:100 in 0.2% PBS-T containing 2.5% DMSO and 1% NDS. After three 1 h washes and an overnight wash in 0.2% PBS-T containing 3% NaCl**,** samples were incubated for 48 h at 4 °C with Alexa 647 anti-goat secondary antibody (Jackson ImmunoResearch) diluted at 1:250 in 0.2% PBS-T containing 2.5% DMSO and 1% NDS. Following identical washing conditions, spinal cords were transferred into RapiClear® 1.49 (SunJinLab) for optical clearing for 24 h at 37 °C and glued ventral side down to a glass-bottom dish filled with RapiClear® 1.49. After imaging, spinal cords were removed from the dish and incubated overnight in PBS to reverse clearing. The next day, they were cut at a vibratome into transverse serial sections and mounted directly onto slides in a consecutive manner with PBS:Glycerol (3:7) for final imaging. Similar procedures regarding immunofluorescence and clearing were applied to perfused L1 segments with additional HB9 staining (diluted 1:3000) for whole-mount MN segmentation and counting with Cellpose.

### Confocal microscopy and image analysis

Imaging of tissues was conducted using a Leica SP8, Zeiss LSM 700, or Olympus FV300 inverted confocal microscope. Sectioned spinal cords were imaged using either a 10× objective for hemisection overviews, a 20× objective for MN pool images, with a z-step size of 4 µm between optical planes, or a 63× objective for close-up images. Whole-mount lumbar spinal cords and isolated L1 segments were scanned using a 25× objective at a z-step size of 1 µm. NMJs were imaged using a 20× objective and a z-step size of 4 µm.

For retrogradely filled spinal cords, segment borders were determined from retrograde labeling for both whole-mount imaging and after sectioning. Only ChAT+ MNs located in the ventral horn and containing a visible nucleus were counted, to avoid double-counting from adjoining sections. For all vibratome sectioned spinal cord segments the following numbers of sections were used to quantify MNs from: C57BL/6 L1-L6 P4 7 sections, L1 P14 9 sections, P28, P42, P64 and P180 14 sections; SMN∆7 and controls L1 P4 10 sections, P9 12 sections and P13 13 sections, C6, T9, L1-L5 P10 10 sections and L6 P10 8 sections; Type III-like SMA and P180 C6, T9, L1, L5 P180 14 sections; SOD1-G93A and SOD1-WT C6, T9, L1, L5 P130 14 sections. For cryostat-sectioned spinal cord segments, every third section was analyzed to further prevent duplicate counting of the same MNs. When applicable, NeuN+/ChAT+ MNs were classified as α-MNs, whereas ChAT+ MNs lacking NeuN immunoreactivity were classified as γ-MNs. If stated, Abercrombie correction was applied the following way: $\mathrm{N}= \frac{\mathrm{n}*\mathrm{T}}{(\mathrm{T}+\mathrm{D})}$ with N = corrected number of MNs, n = original counted number of MNs, T = section thickness in µm, D = mean diameter of MN nucleus in µm.

For analysis of the other MN markers HB9, Nissl and SMI-32 in comparison to ChAT, confocal images were acquired with a 10× objective. MN pools of each analyzed slice were measured in length and width according to ChAT signaling and the mean plus 3 times the SD width and length was calculated from the data acquired from 5 slices within each mouse. This calculated width and length were then applied to the corresponding MN pools for analysis. To guarantee that all MNs were counted, we used the previously reported mean γ-MN area (232.4 µm^2^; [45]) and subtracted three SD, yielding 82.4 µm^2^, with all marker‑positive signals exceeding this area included in the analysis. The amount of marker+ MNs (ChAT +) was calculated as well as the amount of putative MNs that were ChAT- but marker+.

To quantify soma size, the soma perimeter was outlined in the z plane showing the largest nuclear cross section, and the enclosed area was measured in µm^2^. For MN soma size quantification across mouse models and human spinal cords, at least five sections per animal or 3 for human spinal cords were analyzed using ChAT immunoreactivity to delineate the soma boundary. For the comparative analysis of marker-positive ChAT+ and ChAT- cells, two sections per animal were analyzed; ChAT immunoreactivity was used to delineate soma boundaries of ChAT+ cells, whereas the respective marker channel was used for ChAT- cells. For all analyses, either Leica Las X software or Fiji Image-J software was used.

### Training and implementation of MN quantification with cellpose software

Confocal z-stack images of whole-mount L1 spinal cord segments from control and SMN∆7 mice, immunolabeled for ChAT and HB9, were preprocessed in FIJI (ImageJ) to optimize Cellpose-based counting. For the HB9 channel, 3D stacks were despeckled, median-filtered (radius 0.3 pixels), background-subtracted (rolling ball radius 60 pixels), and histogram-equalized to normalize contrast. For ChAT, brightness and contrast were adjusted. The TissueNet model in Cellpose was chosen as the base and further trained with human-in-the-loop annotation using five representative 2D images from both control and SMN∆7 samples [54, 55].

After training, the optimized model was applied to the corresponding 3D stacks to segment ChAT+ MNs using HB9 nuclear signal as an auxiliary marker and generate automated counts. These automated MN counts were directly compared to manual counts obtained by manual visual inspection of the same image stacks. Segmentation served solely as an intermediate detection step for enumeration; no pixel-level segmentation accuracy was assessed, as the aim was to compare MN counts between the two methods. To quantify correspondence between automated and manual counts, precision was calculated as: $\mathrm{P}\mathrm{r}\mathrm{e}\mathrm{c}\mathrm{i}\mathrm{s}\mathrm{i}\mathrm{o}\mathrm{n}= \frac{\mathrm{T}\mathrm{P}}{\mathrm{T}\mathrm{P}+\mathrm{F}\mathrm{P}+\mathrm{F}\mathrm{N}}$ where true positives (TP) were MNs identified by both manual counting and Cellpose segmentation, false positives (FP) were MNs segmented by Cellpose but not counted manually, and false negatives (FN) were MNs counted manually but missed by Cellpose. This definition of precision is adapted here to reflect agreement in MN counting rather than classical pixel-wise segmentation performance metrics.

### Randomization approach for sampling from spinal segments

MN counts per vibratome section for individual lumbar segments (L1–L6) were obtained from n = 4 control and SMN∆7 mice, as described above. These datasets were used as input for a custom R script (R Foundation for Statistical Computing, Vienna, Austria) to evaluate how different randomized sampling strategies, similar to those commonly applied in the field, affect the detection of MN loss. The code is available at GitHub: [URL]: https://github.com/GerstnerF/Counting-Simulation.git. In each simulation run, sampling points were randomly selected to simulate the following approaches: drawing five consecutive sections from anywhere in the lumbar region, selecting every 2nd or every 10th section through the entire lumbar cord starting at a random position in L1, or selecting five random sections within L1 or L2 separately. The R script was executed four times for each sampling scheme, with each execution generating a new independent random selection (“simulation round”). For every round, the resulting subsets from the control and SMN∆7 datasets were used to calculate mean MN counts per genotype, and the values were statistically compared to determine whether each strategy could detect significant MN loss.

### Quantification and statistical analysis

All experimental results are expressed using a calculated mean and bounded by the standard error of the mean (SEM). In this manuscript, n refers to the number of patients or mice used in each group. Each experiment included at least three biological replicates per group. To assess the symmetry of data distribution, the Shapiro–Wilk normality test was applied. For comparisons between two groups, statistical analysis depended on the data’s distribution and the equality of standard deviations (SDs). If the data followed a parametric distribution and the SDs were equal, an unpaired t-test was used. When the SDs were unequal, Welch’s t-test was applied. For nonparametric distributions, the Mann–Whitney test was performed. For comparisons among three groups or more, a one-way ANOVA followed by Tukey’s multiple comparison test was used for parametric distributions. Nonparametric distributions were analyzed using the Kruskal–Wallis test with Dunn’s correction. When data was compared against a set value and the distribution was parametric, a one-sample t-test, when the distribution was non-parametric, a Wilcoxon signed-ranked test was performed. Soma area data were binned and plotted as frequency distributions (%). For MN soma size quantification across mouse models, data were binned at 25 µm^2^, and for human spinal cord data bins of 50 µm^2^ were used. For the comparative analysis of marker-positive ChAT+ and ChAT- cells, soma area distributions of ChAT+ cells were fitted using a sum of two Gaussian distributions to capture the bimodal size distribution reflecting α- and γ-MN populations, and a lognormal distribution for ChAT- cells. For all other distributions, Gaussian and lognormal fits were compared and the model with the higher probability of being correct was selected. Goodness of fit was assessed by R^2^, which exceeded 0.67 for mouse tissue and 0.53 for human tissue across all fitted distributions. The statistical tests used for each experiment are indicated in the respective figure legends. All statistical analyses were conducted using GraphPad Prism 10, and p-values are reported within the figures.

## Supplementary Information


Supplementary Material 1: Figure S1. Additional analyses of the meta-analysis using Bayesian and frequentist models. (A) Distribution of SMA mouse models across studies included in the SLR (%). (B) Distribution of spinal cord regions analyzed across studies in the SLR (%). (C) Distribution of MN markers used across studies in the SLR (%). (D) Pairwise comparisons between subgroups. SMD: standardized mean difference, representing the relative effect size of a given group compared to a reference group. Positive values indicate less MN loss relative to the comparison group. CI: confidence interval. (E) Bayesian pairwise contrasts between subgroups. PMD: posterior mean difference, expressed as Hedges’ g. Positive values indicate less MN loss in a given group relative to the comparison group. CrI: credible interval; PP: posterior probability; ER: evidence ratio, defined as the ratio of the probability of a true difference between subgroups (H1) to the probability of no difference (H0). ER < 1 indicates evidence in favor of H0, whereas ER > 1 indicates evidence in favor of H1.(F) Model fit metrics for multilevel meta-analysis and subgroup analysis. LogLik: log-likelihood, indicating goodness of fit (higher values indicate better fit). AIC: Akaike information criterion; BIC: Bayesian information criterion. Lower AIC and BIC values indicate better model fit and parsimony. Figure S2. Experimental approaches for spinal cord clearing, retrograde MN labeling, and dissection. (A) Images of a perfused C57BL/6 P120 mouse during spinal cord dissection. The left image shows the spinal column after removal of internal organs, the middle image shows the body after ventral access to the spinal canal, and the right image shows the isolated spinal cord with intact, partially pinned ventral roots. Ventral roots corresponding to T9 and L1–L6 are indicated. The lower image shows a higher magnification of the right panel. Scale bars = 500 µm. Yellow and green arrows indicate ventral roots L5 and L6, respectively. (B) Images of a C57BL/6 spinal cord at P4 before (left) and after (right) clearing with RapiClear® 1.49. Scale bars = 250 µm. (C) DIC image of a C57BL/6 spinal cord at P4 during retrograde backfilling to label MNs via L1–L6 ventral roots (VR), using alternating fluorescent dextran dyes (Texas Red and Fluorescein Dextran). Scale bar = 500 µm. (D) DIC and fluorescent images of the spinal cord after retrograde ventral root labeling with Texas Red (magenta) and Fluorescein Dextran (green). Scale bar = 500 µm. (E) Immunostaining of a C57BL/6 P4 L2 segment for ChAT (gray), showing retrogradely labeled MNs with Fluorescein Dextran (green). Scale bar = 20 µm. (F) Quantification of retrogradely labeled L1 MNs positive for ChAT, expressed as a percentage in SMNΔ7 control P4 mice (n = 3). (G) Quantification of ChAT+ MNs positive for retrograde dye, expressed as a percentage in SMNΔ7 control P4 mice (n = 3). (H) Immunostaining of the L1 segment for ChAT (green) in P4, P14, and P42 C57BL/6 mice. Scale bar = 50 µm. (I–K) Quantification of MNs in the L1 segment of SMNΔ7 control mice (n = 3) at P4, P9, and P13 following retrograde ventral root labeling. (I) Number of MNs per vibratome section (75 µm), (J) total number of sections in the L1 segment, and (K) total MN number. This approach is similar to Approach 2 but without initial clearing and reversal steps. MNs were quantified based on ChAT immunoreactivity (not shown). Data in (F, G, I–K) are partially adapted from 26. Statistical analysis was performed using one-way ANOVA (I, K). Figure S3. Marker specificity, soma size distributions, and fixation effects on motor neuron labeling in mouse spinal cord. (A) Immunostaining of L5 hemicord (upper row) and MN pool (lower row) for ChAT (green), SMI-32, Nissl, and HB9 (magenta) from a C57BL/6 mouse at P42. Scale bars = 100 µm. Yellow asterisks indicate marker- ChAT+ MNs, and yellow arrows indicate marker+ ChAT- putative MNs. MN outlines, as identified by ChAT, are shown as white dotted outlines. (B, C) Quantification of SMI-32, Nissl, and HB9 labeling in L5 within ChAT+ MNs (B) and in ChAT- cells (putative MNs) (C), expressed as percentages within the ventral horn surrounding the MN pool of P42 C57BL/6 mice (n = 3 for HB9; n = 5 for SMI-32 and Nissl), as indicated by the white box in (A). (D) Frequency distribution (%) of cells labeled with ChAT and either HB9, SMI-32, or Nissl and ChAT- cells labeled with Nissl and SMI-32 only, plotted against cell cross-sectional area (µm²) and binned at 25 µm² from L1 of P42 C57BL/6 mice. Cells were analyzed within the ventral horn surrounding the MN pool as indicated by the white box in Fig. 3A. Curves represent sum of two Gaussian fits for ChAT+ cells, and lognormal distributions for ChAT- cells (n = 4). (E, F) Quantification of average MN (ChAT+ marker+) area (E) and putative MN (ChAT- marker-) area (F) cross-sectional area (µm²) from the data shown in (D). (G) Immunostaining of L1 for ChAT (green), HB9 (magenta), and NeuN (blue) from a C57BL/6 mouse at P42. Scale bar = 50 µm. ChAT+/NeuN- cells correspond to γ-MNs, whereas ChAT+/NeuN+ cells correspond to α-MNs. Yellow arrows indicate HB9+ γ-MNs. (H) Immunostaining of L1 for ChAT, HB9, and SMI-32 (magenta) in ChAT-eGFP (green) mice at P10 and P60, following dissection in cooled, oxygenated ACSF and subsequent PFA immersion fixation. Scale bar = 50 µm. Statistical analysis was performed using a Wilcoxon signed-rank test against ChAT+ MN values (B), a Kruskal–Wallis test (C, E) or unpaired t-test (F). Figure S4. A nuclear marker facilitates Cellpose-based segmentation. (A) Quantification of L1 MN nuclear diameter (µm) in sections cut using a vibratome (75 µm) or cryostat (20 µm) in P10 SMNΔ7 and control mice (for both sectioning methods: control, n = 4; SMA vibratome, n = 3; SMA cryostat, n = 5). (B) Custom-made chamber for pinning spinal cords for whole-mount immunostaining, clearing, and confocal imaging. T13–L5 ventral roots are pinned. Scale bar = 4 mm. (C) Immunostaining of cleared whole-mount L1 MN pool for HB9 (magenta) in a C57BL/6 mouse at P4, segmented using Cellpose (yellow outlines) based on ChAT labeling (not shown). Scale bars = 50 µm and 20 µm. Statistical analysis was performed using a Kruskal–Wallis test. Figure S5. MN loss in proximal segments can be reproduced using a random sampling simulation approach. Results of randomization simulations of MN number per slice in P10 control versus SMNΔ7 mice for five randomly selected slices from L2 (n = 4 per group). Four simulation rounds are shown (1–4). Statistical analysis was performed using an unpaired t-test for each round. Figure S6. Pronounced disease-related changes in MN size. (A) High-magnification confocal image of L1 control and SMNΔ7 MNs labeled with ChAT (green) and NeuN (blue) at P10. White dotted lines indicate α-MN soma outlines, and yellow dotted lines indicate γ-MN soma outlines. Scale bar = 20 µm. (B) Frequency distribution (%) of P10 L1 control and SMNΔ7 α- and γ-MNs plotted against MN cross-sectional area (µm²), binned at 25 µm². Curves represent lognormal distribution fits (n = 4). (C, D) Quantification of average α-MN (C) and γ-MN (D) cross-sectional area (µm²) from the data shown in (B). (E) High-magnification confocal image of MNs from the thoracic spinal cord of human control (CNTRL) and SMA samples labeled with ChAT (green) and NeuN (blue). White dotted lines indicate α-MN soma outlines, and yellow dotted lines indicate γ-MN soma outlines. Scale bar = 20 µm. (F) Frequency distribution (%) of thoracic human control and SMA α- and γ-MNs plotted against MN cross-sectional area (µm²), binned at 50 µm². Curves represent lognormal distribution fits, except for control α-MNs, which follow a Gaussian distribution (control: n = 3; SMA: n = 5). (G, H) Quantification of average α-MN (G) and γ-MN (H) cross-sectional area (µm²) from the data shown in (F). (I–L) Quantification of MN pools in C6 (I), T9 (J), L1 (K), and L5 LMC and MMC (L) spinal segments of P180 control and Type III-like SMA mice (n = 4 per group, except L5: n = 6). (M) Immunostaining of L1 for ChAT (green), NeuN (blue), and p53 (red) in control and Type III-like SMA mice at P270. Scale bar = 50 µm. (N) Immunostaining of tibialis anterior (TA) and quadratus lumborum (QL) muscles for 2H3 and SV2 (green, presynaptic) and BTX (magenta, postsynaptic) in control and Type III-like SMA mice at P270. Scale bar = 50 µm. (O, P) Quantification of innervated NMJs (%) in TA (O) and QL (P) muscles from the same groups as in (N) (n = 3). (Q) High-magnification confocal image of L5 MNs from SOD1-WT and SOD1-G93A mice labeled with ChAT (green) and NeuN (blue) at P130. White dotted lines indicate α-MN soma outlines, and yellow dotted lines indicate γ-MN soma outlines. Scale bar = 20 µm. (R) Frequency distribution (%) of P130 L5 SOD1-WT and SOD1-G93A α- and γ-MNs plotted against MN cross-sectional area (µm²), binned at 25 µm². Curves represent lognormal distribution fits, except for SOD1-WT α-MNs, which follow a Gaussian distribution (n = 4). (S, T) Quantification of average α-MN (S) and γ-MN (T) cross-sectional area (µm²) from the data shown in (R). Statistical analysis was performed based on animal means using unpaired t-test (C, D, G, H, I–L, S, T), Mann–Whitney test (O, J α-MNs, L γ-MNs), Welch’s t-test (K γ-MNs, L α-MNs), or one-sample t-test (P).
Supplementary Material 2.
Supplementary Material 3.


## Data Availability

The authors confirm that the data supporting the findings of this study are available within the article and its Supplementary material. Some data are not publicly available owing to patient-related restrictions, because they contain information that could compromise the privacy of research participants. Additionally, certain derived data from mouse experiments are available from the corresponding authors upon reasonable request.
