# Supplementary material for: A standardized framework resolves ambiguity in motor neuron loss across neurodegenerative diseases
Source: Acta Neuropathol Commun. 2026 Sep 1;14:182. doi: 10.1186/s40478-026-02415-7 (PMC13536862; doi:10.1186/s40478-026-02415-7)
Supplement: Supplementary file 1 [file 40478_2026_2415_MOESM1_ESM.pdf]

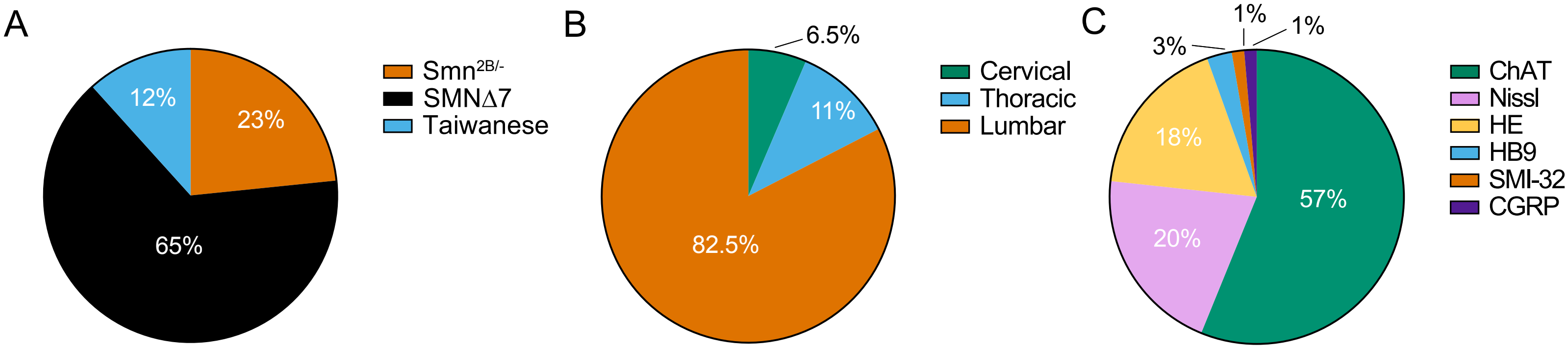

D

Comparison	ESMD	CI	p-value
L3-L6 vs L5 Med	0.92	[-0.35,2.18]	0.152
L3-L6 vs Lumbar	0.16	[-0.80,1.12]	0.734
L5 Med vs Lumbar	-0.76	[-1.99,0.46]	0.221
L3-L6 vs L1-L2	2.00	[1.03,2.98]	<0.0001
L5 Med vs L1-L2	1.08	[-0.14,2.30]	0.0828
Lumbar vs L1-L2	1.84	[0.97,2.70]	<0.0001

E

Comparison	PMD	CrI	PP	ER
L3-L6 vs L5 Med	0.78	[-0.35,1.91]	0.665	1.98
L3-L6 vs Lumbar	0.23	[-0.54,1.00]	0.860	6.16
L5 Med vs Lumbar	-0.56	[-1.61,0.49]	0.764	3.24
L3-L6 vs L1-L2	1.90	[0.90,2.91]	0.992	124
L5 Med vs L1-L2	1.12	[-0.12,2.34]	0.494	0.976
Lumbar vs L1-L2	1.67	[0.77,2.58]	0.989	91

F

Comparison	LogLik	AIC	BIC
Multilevel Model	-95.60	197.20	203.11
Subgroup Model	-82.74	177.48	188.95

Suppl. Figure 1

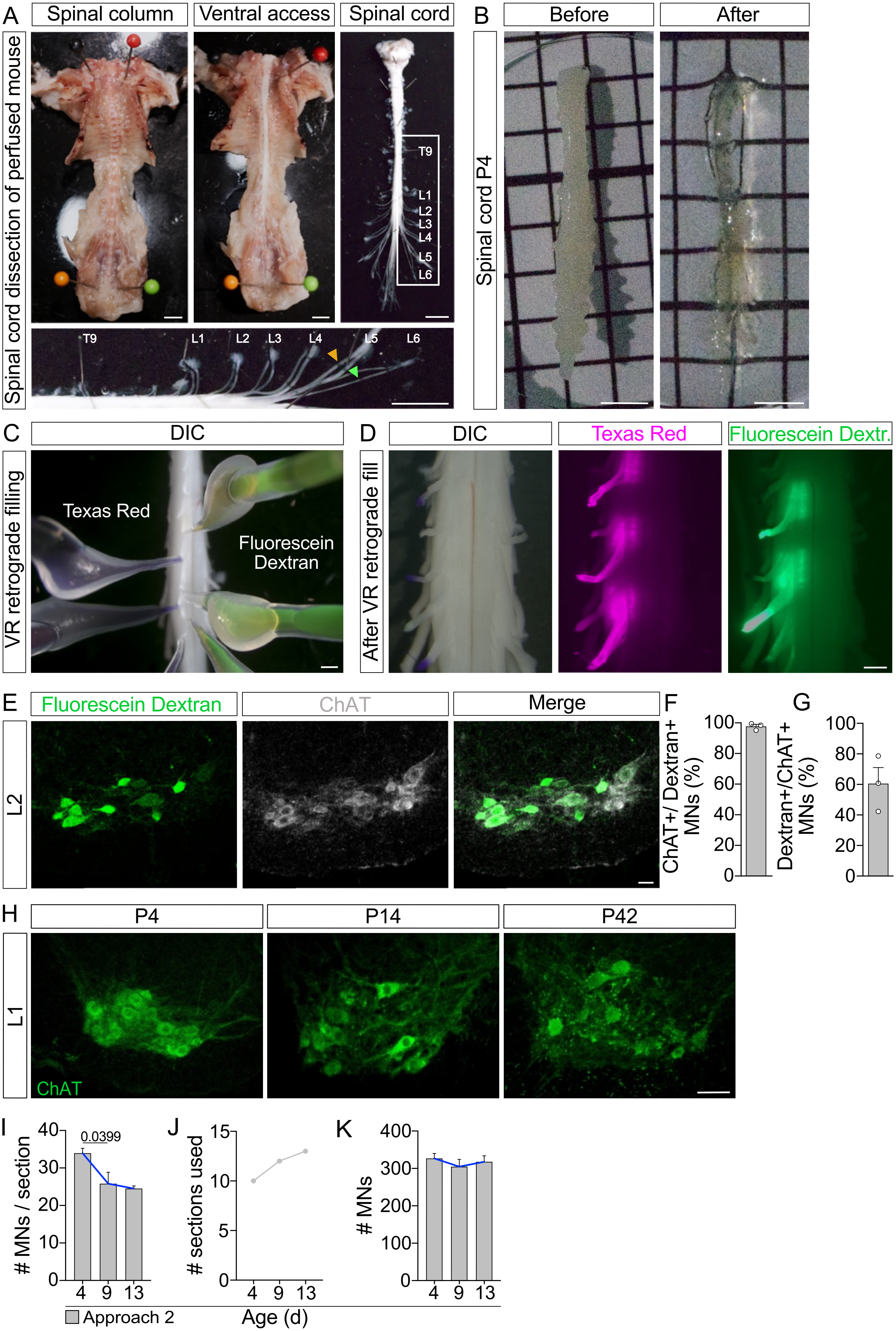

Suppl. Figure 2

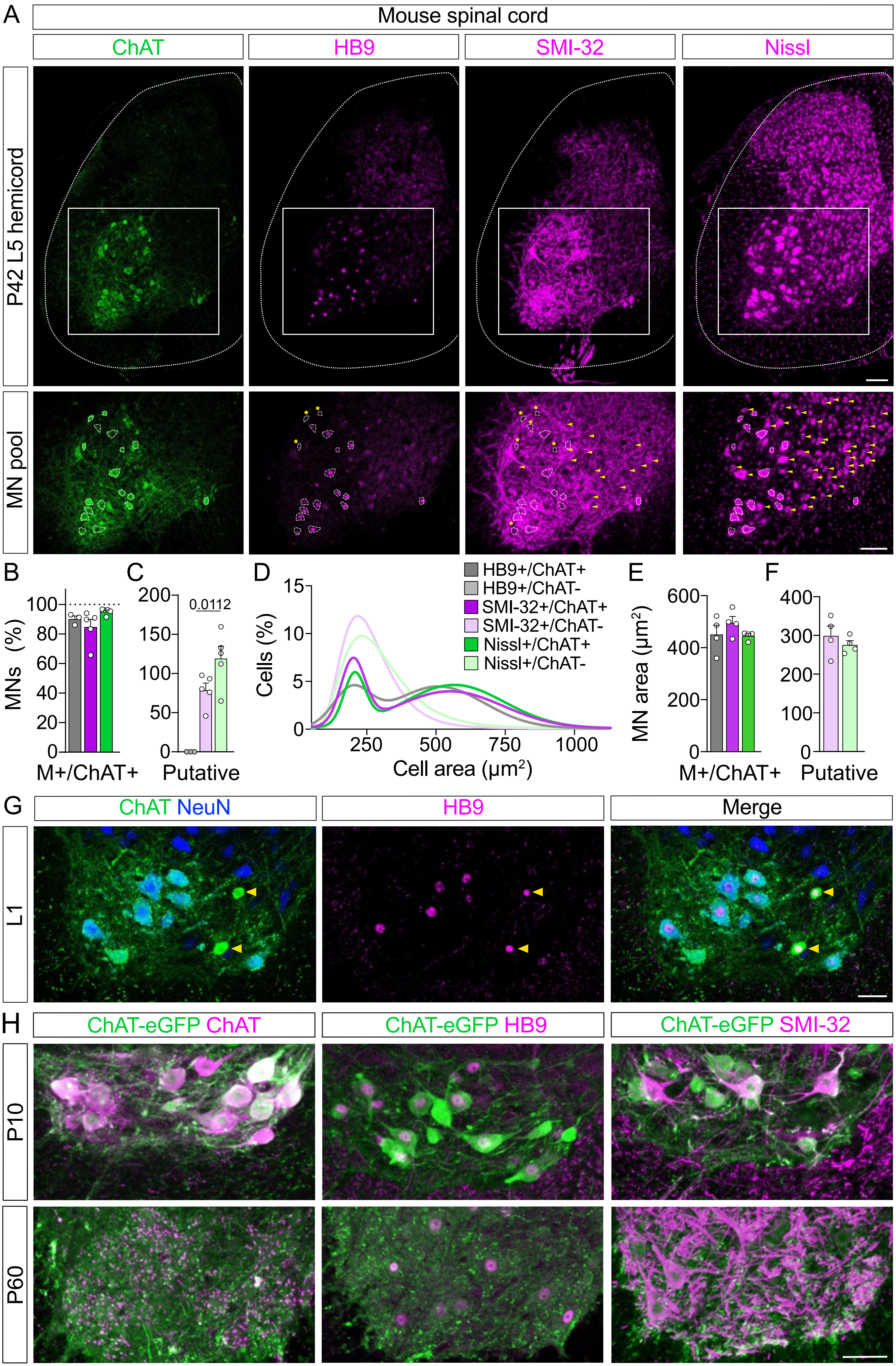

Suppl. Figure 3

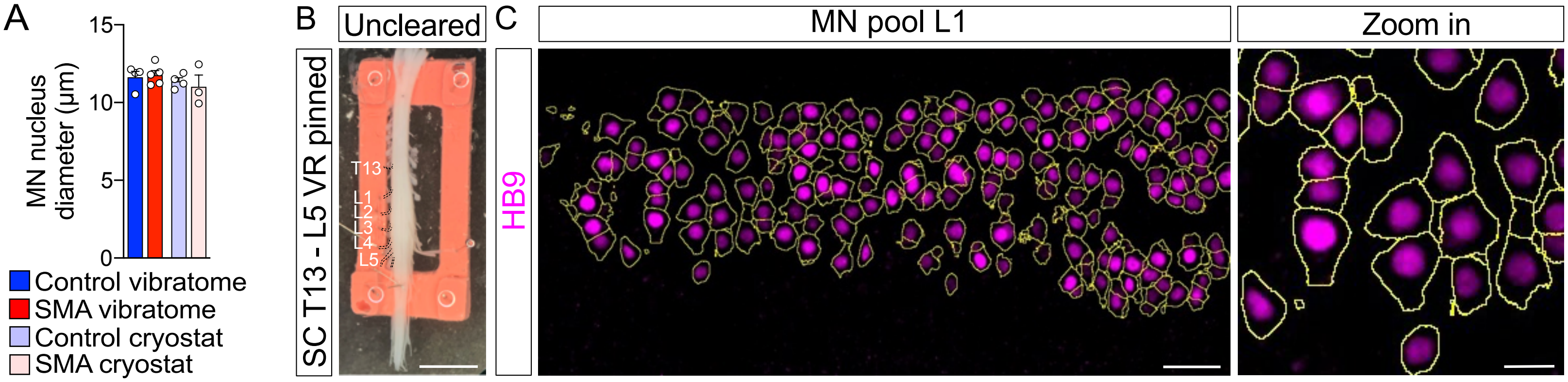
A
MN nucleus diameter (µm)
15
10
5
0
Control vibratome
SMA vibratome
Control cryostat
SMA cryostat
B
Uncleared
SC T13 - L5 VR pinned
T13
L1
L2
L3
L4
L5
C
MN pool L1
Zoom in
HB9

A

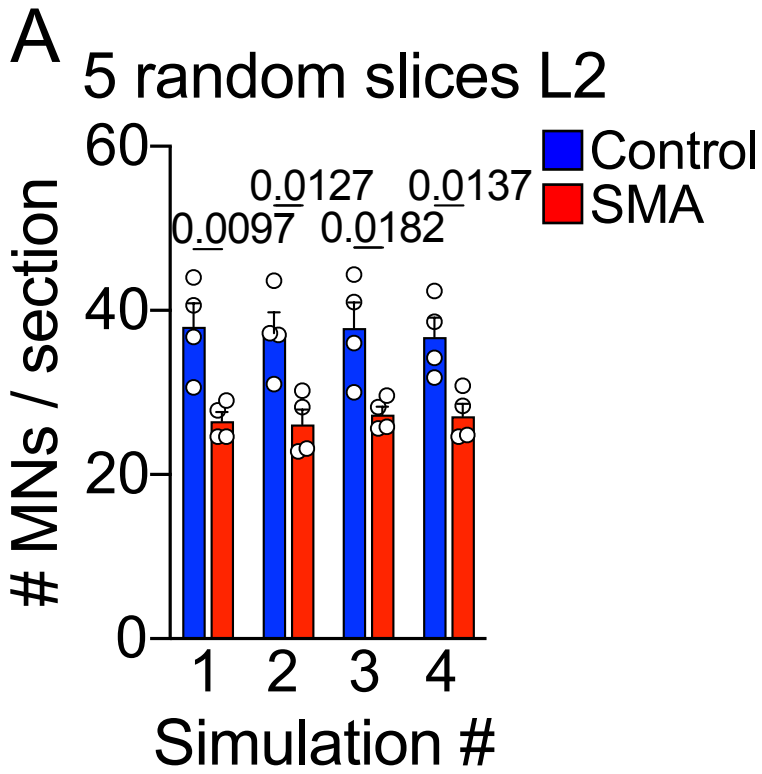

5 random slices L2
Control
SMA
0.0097
0.0127
0.0182
0.0137
MNs / section
60
40
20
0
1
2
3
4
Simulation #

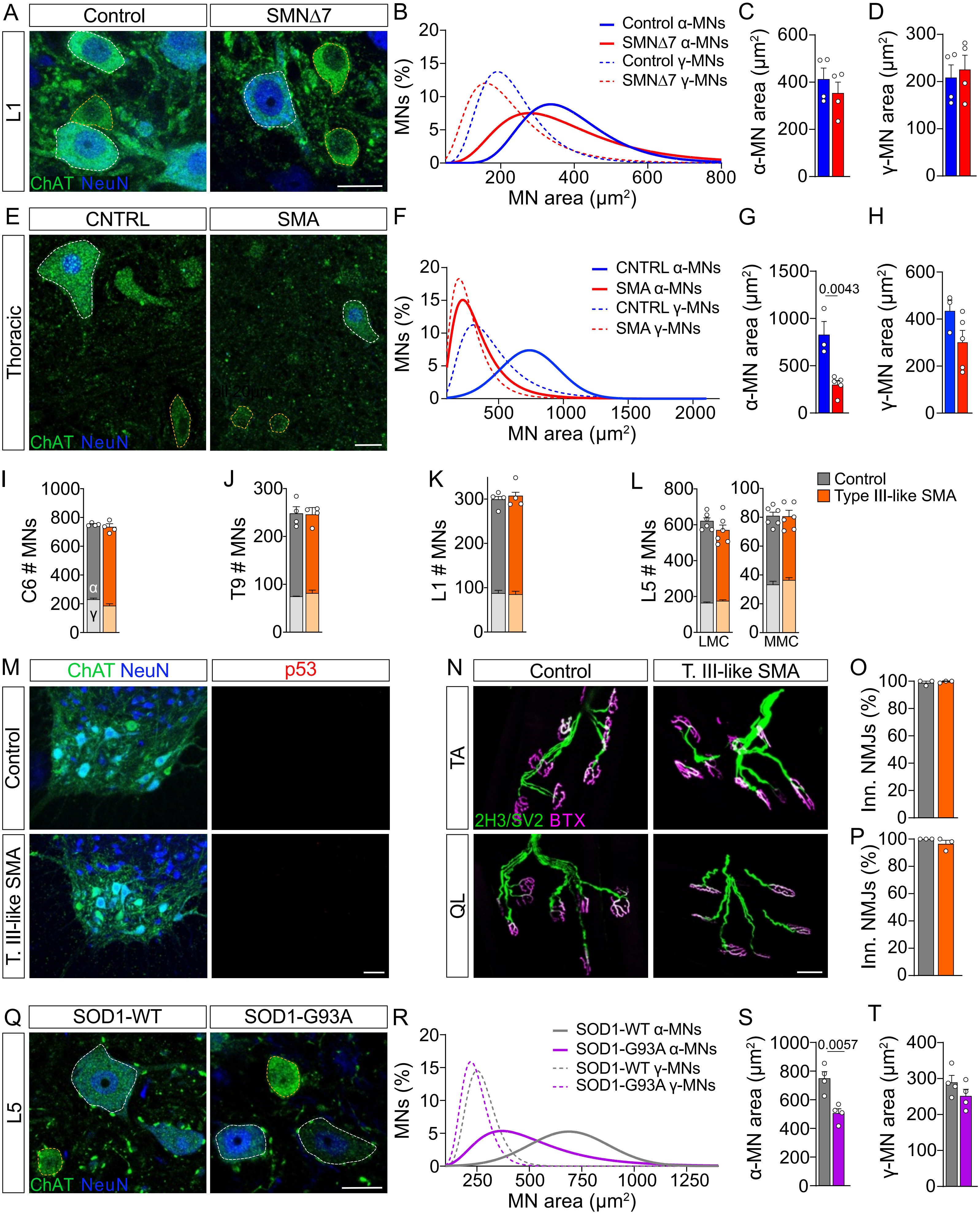

Suppl. Figure 6
