# Supplementary material for: A standardized framework resolves ambiguity in motor neuron loss across neurodegenerative diseases
Source: Acta Neuropathol Commun. 2026 Sep 1;14:182. doi: 10.1186/s40478-026-02415-7 (PMC13536862; doi:10.1186/s40478-026-02415-7)
Supplement: Supplementary file 2 [file 40478_2026_2415_MOESM2_ESM.pdf]

Table S1: PubMed Search Strategy

Step	Terms
1	(atrophy, spinal muscular[MeSH Terms]) OR (spinal muscular atrophy[Title/Abstract]) AND ((2005/1/1:2024/2/20[pdat]) AND (english[Filter]))
2	(murine[Title/Abstract] OR mouse[Title/Abstract] OR mice[Title/Abstract] OR rodent*[Title/Abstract]) AND ((2005/1/1: 2024/2/20 [pdat]) AND (english[Filter]))
3	1+2
4	(((((((SMNΔ7) OR (Δ7)) OR (delta-7)) OR (SMND7)) OR (Smn2B/-)) OR (Taiwanese)) OR "SMA mouse model"[Title/Abstract]) OR "SMA mice"[Title/Abstract] AND (2005/1/1: 2024/2/20 [pdat]) AND (english[Filter])
5	3+4

Table S2: PICOS Criteria

Criteria	Inclusion	Exclusion
**Population**	SMNΔ7: Background must be either Jackson 5025 (FVB.Cg-*Grm7*$^{Tg(SMN2)89Ahmb}$ *Smn1*^{tm1Msd} Tg (SMN2*Δ7) 4299Ahmb/J or state a genetic background of Smn/; SMN2/SMN2; SMNΔ7/ SMNΔ7. The age is between or including P7 and P14.<br><br>Smn$^{2B/-}$: Background must show either a *Smn*$^{2B/2B}$ × *Smn*$^{+/-}$ or state reception from Kothary Lab. The age is between P10 and P28.<br><br>Taiwanese: Background contains 2 copies of SMN gene with a genetic background of Jackson 5058 (FVB.Cg-*Smn1*^{tm1Hung} Tg(SMN2)2Hung/J) or *Smn* $^{-/-}$ *; SMN2* $^{tg/0}$. The age of the two copy model is between P7 and P14.	All mouse models not matching this criteria or altered genetically/cellularly in any further manner.
**Intervention**	Studies provide data for a non treated experimental mouse model.	Studies provide no data for a non treated experimental mouse model.
**Comparison**	Studies provide data for a non treated control mouse model.	Studies provide no data for a non treated control mouse model.
**Outcomes**	MN counts from the anterior spinal cord are reported or a percentage of loss is described.	MN counts in cell culture or not within the spinal cord anterior horn.
**Study Design and Publication Type**	Four-arm parallel-group randomized control studies and comparative studies were evaluated.	All other study types.

Table S3: PRISMA Diagram

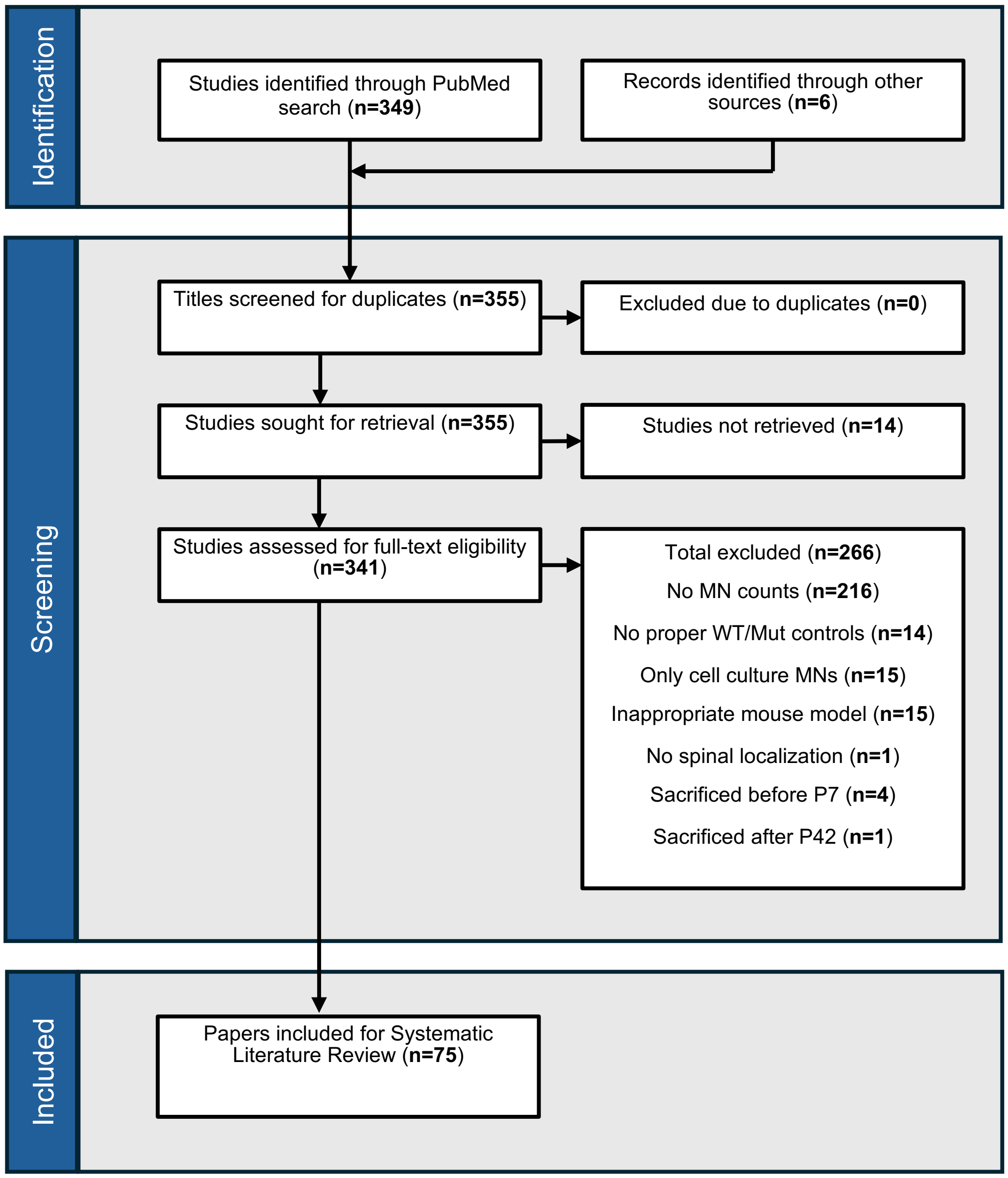

Table S4: List of patient tissue used for immunohistochemical analysis

Patient ID	SMA Type	Age	PMI	Analyzed properties
CNTL 12_04	Control	5 years	21 h	MN marker
CNTL 12_06	Control	3 years	~2 h	MN size
CNTL 13_01	Control	14 years	2 h	MN size, MN marker
CNTL 17_01	Control	9 months	14-18 h	MN size, MN marker
SMA 10_01	Type 1	14.8 months	< 2 h	MN size
SMA 14_04	Type 1	6 years	2 h	MN size
SMA 14_05	Type 1	8 months	3-9 h	MN size
SMA 17_04	Type 1	24 months	2 h	MN size
SMA 19_01	Type 1	11 months	19 h	MN size

Table S5: List of primary antibodies

Name	Company	Cat #	Host	Dilution	Labeled Structure
Bungarotoxin	Invitrogen	B35451	N/A	1:500	NMJ (post-synaptic)
Calbindin	Synaptic Systems	214006	Chicken	1:500	Renshaw cells
ChAT	Millipore	AB144P	Goat	1:500	MNs
FoxP2	Abcam	ab16046	Rabbit	1:5000	V1 interneurons (nucleus)
GFP	Abcam	ab13970	Chicken	1:2000	GFP+ MNs
HB9	Custom made (Wichterle Lab)		Guinea pig	1:5000	MNs (nucleus)
Nissl NeuroTrace 500/525	Invitrogen	N21480	N/A	1:500	MNs
NeuN	Millipore	MAB377	Mouse	1:2000	α-MNs
Neurofilament	DSHB	2H3, concentrate	Mouse	1:500	NMJ (pre-synaptic)
Neurofilament-A647 (SMI-32)	Biolegend	801712	Mouse	1:500	MNs
p53	Leica	NCL-Lp53-CM5p	Rabbit	1:1000	Nucleus
SV2	DSHB	SV2, concentrate	Mouse	1:500	NMJ (pre-synaptic)
